# Drought increases heat tolerance of leaf respiration in *Eucalyptus globulus* saplings grown under both ambient and elevated atmospheric [CO_2_] and temperature

**DOI:** 10.1093/jxb/eru367

**Published:** 2014-09-09

**Authors:** Paul P. G. Gauthier, Kristine Y. Crous, Gohar Ayub, Honglang Duan, Lasantha K. Weerasinghe, David S. Ellsworth, Mark G. Tjoelker, John R. Evans, David T. Tissue, Owen K. Atkin

**Affiliations:** ^1^Division of Plant Sciences, Research School of Biology, Building 46, The Australian National University, Canberra, ACT, 0200, Australia; ^2^Department of Geosciences, Princeton University, Guyot Hall, Princeton, NJ 08544, USA; ^3^Hawkesbury Institute for the Environment, University of Western Sydney, Hawkesbury Campus, Locked Bag 1797, Penrith, NSW, 2751, Australia; ^4^Department of Horticulture, Agricultural University Peshawar, 25130, Khyber Pakhtunkhwa, Pakistan; ^5^Institute of Ecology & Environmental Science, Nanchang Institute of Technology, No. 289 Tianxiang Road, Nanchang 330099, China; ^6^Faculty of Agriculture, University of Peradeniya, Peradeniya, 20400, Sri Lanka; ^7^ARC Centre of Excellence in Plant Energy Biology, The Australian National University, Canberra, ACT, 0200, Australia

**Keywords:** Dark respiration, drought, elevated CO_2_, *Eucalyptus globulus*, Q_10_, temperature response.

## Abstract

Climate change is resulting in increasing atmospheric [CO_2_], rising growth temperature (*T*), and greater frequency/severity of drought, with each factor having the potential to alter the respiratory metabolism of leaves. Here, the effects of elevated atmospheric [CO_2_], sustained warming, and drought on leaf dark respiration (*R*_dark_), and the short-term *T* response of *R*_dark_ were examined in *Eucalyptus globulus.* Comparisons were made using seedlings grown under different [CO_2_], *T*, and drought treatments. Using high resolution *T*–response curves of *R*_dark_ measured over the 15–65 °C range, it was found that elevated [CO_2_], elevated growth *T*, and drought had little effect on rates of *R*_dark_ measured at *T* <35 °C and that there was no interactive effect of [CO_2_], growth *T*, and drought on *T* response of *R*_dark_. However, drought increased *R*_dark_ at high leaf *T* typical of heatwave events (35–45 °C), and increased the measuring *T* at which maximal rates of *R*_dark_ occurred (*T*_max_) by 8 °C (from 52 °C in well-watered plants to 60 °C in drought-treated plants). Leaf starch and soluble sugars decreased under drought and elevated growth *T*, respectively, but no effect was found under elevated [CO_2_]. Elevated [CO_2_] increased the *Q*_10_ of *R*_dark_ (i.e. proportional rise in *R*_dark_ per 10 °C) over the 15–35 °C range, while drought increased *Q*_10_ values between 35 °C and 45 °C. Collectively, the study highlights the dynamic nature of the *T* dependence of *R*_dark_ in plants experiencing future climate change scenarios, particularly with respect to drought and elevated [CO_2_].

## Summary

This study highlights the dynamic nature of the temperature dependence of leaf respiration in plants experiencing future climate change scenarios, particularly with respect to drought and elevated [CO_2_].

## Introduction

Around the world, the frequency and severity of droughts may increase as a result of global climate warming underpinned by rising atmospheric CO_2_ concentrations ([CO_2_]; IPCC, 2013). The average mean temperature of the Earth’s surface is increasing (Rahmstorf *et al.*, 2007), with heatwaves [such as the recent heatwaves in Australia (BOM-Australia, 2014)] predicted to become more common (Meehl and Tebaldi, 2004; Ciais *et al.*, 2005; IPCC, 2013). In C_3_ plants, drought leads to a rapid decrease in photosynthetic carbon gain due to stomatal closure (Hsiao, 1973; Lawlor and Cornic, 2002), with high temperature (*T*) further exacerbating reductions in net carbon gain (Sharkey, 2005; Rennenberg *et al.*, 2006; Hüve *et al.*, 2011); hence, plant productivity is typically lower during hot, dry periods. Collectively, such factors have important consequences for the growth and survival of plants, including economically important species in the forestry industry.

A major factor in determining the productivity and functioning of forest ecosystems is the response of leaf respiration in the dark (*R*_dark_) to changes in the abiotic environment. Of the CO_2_ fixed each day by net photosynthesis in well-watered plants, 20–80% is released back into the atmosphere by plant respiratory processes (Poorter *et al.*, 1990; Atkin *et al*., 1996, 2007; Loveys *et al.*, 2002; Gifford, 2003), with leaves accounting for ~50% of whole-plant *R*_dark_ (Atkin *et al.*, 2007). Small changes in leaf *R*_dark_ (e.g. due to changes in atmospheric [CO_2_], *T*, and/or water availability) could, therefore, have profound effects on functioning of forest ecosystems and the Earth system (Gifford, 2003; King *et al.*, 2006; Atkin *et al.*, 2008; Wythers *et al.*, 2013). Indeed, because leaf *R*_dark_ is *T* sensitive (Atkin *et al.*, 2005; Kruse *et al.*, 2011), several studies have predicted large changes in terrestrial C storage and atmospheric [CO_2_] in a future, warmer world (Cox *et al.*, 2000; King *et al.*, 2006; Huntingford *et al.*, 2013).

In predicting the impacts of future climate change on plant respiration, Earth System Models (ESMs) often assume a constant *Q*_10_ for leaf *R*_dark_ of 2.0 (i.e. *R*_dark_ doubles for every short-term 10 °C rise in *T*) (Huntingford and Cox, 2000) and that *R*_dark_ does not acclimate to sustained changes in growth *T* (Mahecha *et al.*, 2010). While the assumption of a constant *Q*_10_ of 2.0 may be appropriate for modelling rates of *R*_dark_ in some plant species, the assumption is unlikely to be valid for all scenarios, as the *T* response of *R*_dark_ can be highly variable. For example, sustained increases in growth *T* can result in declines in the *Q*_10_ of *R*_dark_ (Atkin *et al.*, 2000*b*, 2005; Zaragoza-Castells *et al.*, 2008), underpinned by limitations in substrate supply and/or energy demand that restrict rates of *R*_dark_ at high measuring *T* more than at low measuring *T* (Atkin and Tjoelker, 2003). Moreover, the *Q*_10_ often declines as measuring *T* increases (James, 1953; Forward, 1960; Tjoelker *et al.*, 2001; Atkin and Tjoelker, 2003; Zaragoza-Castells *et al.*, 2008).

The extent to which the *Q*_10_ of leaf *R*_dark_ declines with increasing measuring *T* varies among species and environments, and is not well understood. However, a ‘generalized’ *Q*_10_–*T* relationship proposed by Tjoelker *et al.* (2001) suggested that *Q*_10_ declines with increasing *T* according to: *Q*_10_=3.09–0.043*T*. Accounting for this *Q*_10_–*T* relationship results in lower *R*_dark_ at *T* both lower and higher than a given reference temperature, leading to large decreases in predicted ecosystem *R*_dark_ compared with models that assume a constant *Q*_10_ of 2.0 (Wythers *et al.*, 2005, 2013). Moreover, variations in the *Q*_10_–*T* relationship due to changes in the environment (e.g. in response to rising atmospheric [CO_2_], growth *T*, and/or drought) that alter the balance between respiratory capacity, substrate supply, and/or energy demand could strongly affect the magnitude of plant *R*_dark_ estimated by ecosystem models and ESMs (Wythers *et al.*, 2005, 2013; King *et al.*, 2006).

Given the link between substrates/energy demand and *Q*_10_ values (Atkin and Tjoelker, 2003), it seems likely that higher substrate supply might result in an increased *Q*_10_ in elevated atmospheric [CO_2_], altering the *T* dependence of *R*_dark_. Similarly, drought-mediated changes in photosynthesis, substrate supply, and energy demand (Ribas-Carbó *et al.*, 2005) could, theoretically, affect the *Q*_10_ of *R*_dark_. In most studies, imposition of drought results in a decline in *R*_dark_ at a set measuring *T* (Flexas *et al.*, 2005; Galmés *et al.*, 2007; Atkin and Macherel, 2009); however, in some cases, drought results in no change (Gimeno *et al.*, 2010) or an increase in *R*_dark_ at set measuring *T* (Zagdanska, 1995; Bartoli *et al.*, 2005; Galmés *et al.*, 2007; Slot *et al.*, 2008; Metcalfe *et al.*, 2010), with one report of drought-mediated increases in the *Q*_10_ of *R*_dark_ (Slot *et al.*, 2008).

Finally, consideration needs to be given to acclimation to increased growth *T* on *R*_dark_ at a set measuring *T* and associated *Q*_10_ values. Acclimation to sustained increases in growth *T* often results in a decline in basal rates of *R*_dark_ (Atkin *et al.*, 2000*a*; Bolstad *et al.*, 2003; Loveys *et al.*, 2003; Tjoelker *et al.*, 2008, 2009; Zaragoza-Castells *et al.*, 2008), that are accentuated by drought (Rodríguez-Calcerrada *et al.*, 2010; Crous *et al.*, 2011). Although sustained changes in growth *T* are reported to have little impact on the *Q*_10_ of *R*_dark_ in some species (Tjoelker *et al.*, 2001; Zaragoza-Castells *et al.*, 2008; Crous *et al.*, 2011), several studies have reported lower average *Q*_10_ values in warm- compared with cold-grown plants (Zha *et al.*, 2002; Armstrong *et al.*, 2008). What is unclear, however, is the extent to which sustained increases in growth *T* impact on *Q*_10_–*T* relationships. The extent to which the *T* dependence of leaf *R*_dark_ is affected by potential interactive effects of atmospheric [CO_2_]–growth *T*–drought is also not known.

Examining how abiotic factors impact on the *T* dependence of leaf *R*_dark_, previous studies either (i) have quantified the impact of diel variations in *T* on leaf *R*_dark_; or (ii) have measured rates of *R*_dark_ at defined *T* intervals (e.g. often every 5 °C) following equilibration of leaves to each *T* interval. While informative, both approaches have their limitations. For example, with approach (i) account needs to be taken of other diel changes, such as changes in irradiance and metabolic functioning of the leaf through the day (Peuke *et al.*, 2013). The quality/resolution of data derived from approach (ii) is often low due to the coarse nature of the measurements, and the fact that such measurements are typically made over a restricted *T* range (e.g. <35 °C), making it difficult to detect significant differences in *Q*_10_–*T* relationships among treatments. Given the limitations of these approaches, an alternative is to record rates of leaf *R*_dark_ as leaves are rapidly heated (e.g. 1 °C min^–1^), following the example of numerous studies assessing thermal tolerance of photosynthesis (Havaux *et al.*, 1991; Knight and Ackerly, 2002; Hüve *et al.*, 2006) and *R*_dark_ (Hüve *et al.*, 2011, 2012; O’Sullivan *et al.*, 2013; Heskel *et al.*, 2014; Weerasinghe *et al.*, 2014). The resultant high-resolution data sets enable the impact of the abiotic treatments on *R*_dark_–*T* curves (and associated *Q*_10_–*T* relationships) to be explored in detail.

At high *T*, leaf *R*_dark_ reaches a maximum (at *T*_max_) at which *Q*_10_=1.0; this point indicates the maximum heat tolerance of *R*_dark_, with further heating resulting in irreversible declines in *R*_dark_ (i.e. *Q*_10_ <1.0), ultimately leading to cell death (Atkin and Tjoelker, 2003; Hüve *et al.*, 2011, 2012; O’Sullivan *et al.*, 2013). Recent studies have reported that the *T*_max_ of leaf *R*_dark_ is near 52 °C in *Phaseolus vulgaris* (Hüve *et al.*, 2012), 51–57 °C in *Eucalyptus pauciflora* (O’Sullivan *et al.*, 2013), 60 °C in several tropical rainforest species (Weerasinghe *et al.*, 2014), and 53–58 °C in an arctic shrub, *Betula nana* (Heskel *et al.*, 2014); these values are markedly higher than the 48 °C value derived from the regression reported in Tjoelker *et al.* (2001). The extent to which leaves can tolerate such *T* is important, as 23% of the Earth’s land surface exhibits maximum air *T* >40 °C (Larcher, 2004), and, in such habitats, sun-exposed leaves can be 10 °C hotter than the surrounding air (Singsaas *et al.*, 1999; Wise *et al.*, 2004), probably resulting in leaf *T* exceeding 50 °C (Hamerlynck and Knapp, 1994; Valladares *et al.*, 2007). Such extremes, while rare now, are likely to become more frequent in the future (Meehl and Tebaldi, 2004; Ciais *et al.*, 2005; IPCC, 2013; Tingley and Huybers, 2013). Here, a crucial factor is the extent to which the *T*_max_ of leaf *R*_dark_ is affected by growth *T*, atmospheric [CO_2_], and/or drought.

Studies on photosynthetic metabolism have reported increased high *T* tolerance in plants subjective to elevated growth *T* (Downton *et al.*, 1984; Seemann *et al*., 1984, 1986; Havaux, 1993), atmospheric [CO_2_] (Faria *et al.*, 1996; Taub *et al.*, 2000), and/or drought (Seemann *et al.*, 1986; Havaux, 1992), with the increased heat tolerance being associated with increases in leaf osmotic potential and soluble sugar concentrations (Seemann *et al.*, 1986; Hüve *et al.*, 2006). Moreover, recent work by Hüve *et al*. (2012) suggests that the *T*_max_ of *R*_dark_ is increased in leaves with enhanced osmotic potential or sugar concentrations (via protection of respiratory membranes). Given this, enhanced concentrations of non-structural carbohydrates in plants grown under elevated atmospheric [CO_2_] (Wullschleger *et al.*, 1992; Tjoelker *et al.*, 1998; Vu *et al.*, 2002; Tissue and Lewis, 2010; Smith *et al.*, 2012; Xu *et al.*, 2012) might be associated with an increase in the *T*_max_ of leaf *R*_dark_. In contrast, environments that lead to depletion of carbon reserves [e.g. elevated growth *T* (Tjoelker *et al.*, 2008) and, in some cases, drought (Adams *et al.*, 2009; Duan *et al.*, 2013; Mitchell *et al.*, 2013)] could potentially lead to a decrease in high *T* tolerance of leaf *R*_dark_, depending on whether plants are grown under ambient or elevated atmospheric [CO_2_] (Hamerlynck *et al.*, 2000; Niinemets, 2010). Importantly, however, no study has yet investigated the impact of multiple climate change drivers on the respiratory *T*_max_.

The overall aim of the present study was to assess how elevated atmospheric [CO_2_], growth *T*, and drought affect the shape of the short-term *T* response of leaf *R*_dark_ (ranging from 15 °C to 65 °C) of a widely distributed, commercially important tree species *Eucalyptus globulus*. The study tested the following hypotheses. First, given that substrates can limit *R*_dark_ (Azcón-Bieto and Osmond, 1983), particularly at high measuring *T* (Atkin and Tjoelker, 2003; Bunce, 2007), and because substrate availability may decrease under conditions of drought/high growth *T*, it was hypothesized that rates of leaf *R*_dark_ at high measuring *T* would be lower in drought-treated plants, with the effects of drought being accentuated by growth of plants under elevated growth *T* and ambient atmospheric [CO_2_] (which increase C turnover and limit CO_2_ uptake, respectively). Further, given the linkage between *Q*_10_ values and substrate supply (Atkin and Tjoelker, 2003), it was hypothesized that exposure to those treatments that reduced soluble sugar concentrations would also be associated with reduced *Q*_10_ values. Finally, given the potential link between the concentration of soluble sugars and high *T* tolerance of *R*_dark_ (Hüve *et al.*, 2012), it was hypothesized that *T*_max_ would be greatest in well-watered plants grown under elevated atmospheric [CO_2_] and ambient growth *T*.

## Materials and methods

### Site description, plant material, and experimental design

The study took place at the Hawkesbury Forest Experiment (HFE) in Richmond, NSW, Australia (33°36’40’’S, 150°44’26.5’’E, elevation 30 m) in a warm humid temperate climate with a mean annual *T* of 17 °C and mean annual precipitation of 800mm. The HFE consisted of 12 CO_2_-, humidity-, and *T*-controlled whole tree chambers (WTCs) surrounded by a continuous block of forest. Two treatments described in further detail in Crous *et al.* (2013) were applied to the WTC: (i) temperatures increased 3 °C above ambient *T*; and (ii) atmospheric [CO_2_] elevated 240 ppm above ambient concentrations, with three replicates per atmospheric [CO_2_] and *T* treatment combination.

A widely planted eucalypt, *E. globulus* Labill., was planted from forestry tube stock seedlings in 5 litre pots in early October 2010 and put in the tree chambers for an 8 week experiment starting on 1 November 2010. Fertilization was applied every week until 8 November 2010, and once more on 3 December 2010. At these times, each pot received ~140ml of 23:4:18 NPK liquid fertilizer containing ~8kg N ha^–1^. Seedlings were ~30cm tall at the start of the experiment and controls grew 3–4cm per week thereafter. Each chamber had six potted seedlings, of which two pots received a well-watered regime (watered daily to field capacity) and four pots received a drought treatment. Drought periods were imposed during weeks 3 and 4 (first drought period; watering reduced from 12 November) and weeks 6 and 7 (second drought period; watering reduced from 4 December) of the experiment by adding only enough water to maintain the stomatal conductance (*g*_s_) between 0 and 100 mmol m^–2^ s^–1^ relative to a well-watered conductance exceeding 500 mmol m^–2^ s^–1^; rewatering pots to full soil water capacity occurred in week 5 to separate the two drought periods. These drought periods are denoted by shaded areas in the relevant figures.

### Leaf respiration and photosynthesis measurements

To monitor plant physiological performance under the different environmental treatments, gas exchange measurements were taken weekly over a 7 week period. For the first drought period, gas exchange measurements commenced on 15 November 2010, while those of the second drought period commenced on 7 December 2010 (i.e. 3 d after onset of both drought periods). Leaves of similar physiological age were measured throughout the experiment representing the most recently fully expanded leaves (i.e. node 3 from the terminal apex on the seedlings). *Eucalyptus globulus* seedlings with juvenile leaves are hypostomatous (i.e. leaves have stomata on their abaxial side only).

Gas exchange measurements were conducted using portable infrared gas analyser (IRGA) systems (LiCor 6400; LiCor Inc., Lincoln, NE, USA) using 6cm^2^ leaf cuvettes. To minimize diffusion gradients across the gaskets of the cuvette (Bruhn *et al.*, 2002; Flexas *et al.*, 2007), CO_2_ levels inside the cuvettes were set to the prevailing [CO_2_] in each WTC. No correction was made for diffusion of water vapour across the gasket (Rodeghiero *et al.*, 2007); however, any error in estimates of light-saturated photosynthesis (*A*_sat_), *g*_s_, and internal CO_2_ concentration (*C*_i_) would have been minor and similar for both well-watered and drought-treated plants in each growth [CO_2_]/growth *T* treatment. Block *T* of the LiCor 6400 was set to the prevailing *T* in each WTC (~18–33 °C; see Supplementary Fig. S1 available at *JXB* online). Measurements were made in the late morning to early afternoon of each sampling day. Photosynthesis was measured at saturating light of 2000 μmol m^–2^ s^–1^ (*A*_sat_). Measurements of leaf *R*_dark_ were made on dark-adapted leaves after being covered with foil for at least 30min to achieve steady-state *R*_dark_ (Azcón-Bieto and Osmond, 1983; Atkin *et al.*, 1998). In week 7, a methodological error occurred when measuring gas exchange under light saturation; therefore, data presented for week 7 are limited to *R*_dark_.

### Temperature–response curves of leaf *R*_dark_

Short-term temperature–response curves of leaf *R*_dark_ for individual leaves from each chamber were measured in well-watered and drought-treated plants between 15 °C and 65 °C in week 7. Temperature control in two custom-made 15.5×11.0×6.5cm water-jacketed, aluminium leaf chambers (each connected to a LiCor 6400) was regulated by a temperature-controlled water bath (Julabo 32HL, Julabo Labortechnik, Seelbach, Germany) and programmed to increase by ~1 °C min^–1^ (O’Sullivan *et al.*, 2013). Within each chamber, two fans mixed air (Micronel, Fellbrook, CA, USA), while leaf *T* was monitored using a small gauge wire copper–nickel–chromium thermocouple (type E) in contact with the abaxial surface of each leaf; the thermocouple was attached to a LI-6400 external thermocouple adaptor (LI640013, Li-Cor Inc.) to enable recording of leaf *T.* The exiting air-stream from each water-jacketed chamber was connected to the ‘sample’ gas line of the LI-6400 [fitted with an empty, sealed 3×2cm cuvette (LI-6400-02B)]. Net CO_2_ exchange (respiration) from the continuously warming, darkened cuvette was calculated via comparison of the ‘sample’ IRGA values with the ‘reference’ IRGA values. Flow rates through the water-jacketed chamber (700 μmol s^–1^) and [CO_2_] of the incoming air were controlled via the LI-6400 console flow meter and LI-6400–01 CO_2_ mixer. Incoming air was fully dried before entering the water-jacketed chamber to ensure that there was no condensation in the sample gas line exiting the water-jacketed chamber (at high leaf *T*, leaves exhibited high rates of water release). The sample and reference gas lines were matched prior to the start of each *T*–response run and several times during the run, with rates of net CO_2_ exchange taking into account dilution of CO_2_ by water vapour.

Short-term *T*–response curves were measured at week 7 on both well-watered and drought-treated plants brought to the lab at least half an hour prior to measurement. In the week prior to the start of the *R*_dark_–*T* analysis, *g*_s_ of each leaf was measured to confirm their drought status (see week 6 values of *g*_s_ in Supplementary Fig. S2 at *JXB* online). To assess leaf area, an image of the leaf was taken before starting the T–response curve and leaves were oven-dried afterwards. Leaf area was determined using Image J Software Analysis (Davidson and PrometheusWikicontributors, 2011).

Previous experiments with another *Eucalyptus* species (*E. pauciflora*) have indicated that short-term *T*–response curves are fully reversible up to 45 °C, but not when irreversible metabolic damage occurred at leaf *T* exceeding 45 °C (O’Sullivan *et al.*, 2013). Given this, modelling of the *T*–response curves (in order to calculate *Q*_10_ values at each leaf *T*) was restricted to the 15–45 °C range. To model *T* responses of leaf *R*_dark_ over the 15–45 °C range, a polynomial equation was used (Atkin *et al.*, 2005; O’Sullivan *et al.*, 2013) fitted to the natural log of *R*_dark_:

$${\text{log}}_{\text{e}}\left(R\right)=a+bT+cT^{\text{2}}$$ (1)

and where:

$$R=e^{a}{}^{+bT+cT^{\text{2}}}$$ (2)

with *T* being leaf *T* (°C) and *a*, *b*, and *c* are coefficients that describe the *T* response of the natural log of *R*, and where *a* represents the natural log of *R*_dark_ at 0 °C. The differential of equation 1 can be used to model the *Q*_10_ of leaf *R*_dark_ at any measuring *T*:

$$Q_{\text{1}0}=e^{\text{1}0\times (b+2cT)}$$ (3)

In past studies using a similar heating protocol, a ‘burst’ in respiration occurred in the ramp up to *T*_max_ (as shown by an inflection point in *R*_dark_ *–T* curves) (Hüve *et al.*, 2011, 2012; O’Sullivan *et al.*, 2013). Such bursts can lead to the activation energies (*E*_a_) of *R*_dark_ being markedly higher above the inflection point (O’Sullivan *et al.*, 2013). They can also result in observed rates of *R*_dark_ exceeding those predicted from curves fitted over a lower range of non-lethal *T*s [e.g. <45 °C (O’Sullivan *et al.*, 2013)].

To assess the effect(s) of growth *T*, [CO_2_], and/or water availability on the magnitude of potential respiratory bursts in *E. globulus*, *E*_a_ values were calculated over two different *T* intervals (each 5–10 °C in range, depending on the *R*_dark_–*T* characteristics of each replicate), which are below *T*_max_ values reported previously (O’Sullivan *et al.*, 2013). For well-watered plants, where *T*_max_ values were ~52 °C, the two intervals were within the 30–40 °C (low range) and 40–50 °C (high range) ranges. For drought-treated plants, where *T*_max_ values were ~60 °C, the intervals were within the 40–50 °C (low range) and 50–60 °C (high range) ranges. In both well-watered and drought-treated plants, the aim was to compare *E*_a_ values over two *T* intervals in the immediate lead up to *T*_max_. In cases where the burst was minimal or non-existent, *E*_a_ values are likely to be lower at the high *T* range compared with the low *T* range (i.e. *E*_a_–high *T*/*E*_a_–low *T* ratios <1); in contrast, where a burst occurs, this ratio was expected to be either near unity or >1.0.

### Leaf carbohydrate analyses

To assess the impact of each growth treatment on the concentration of soluble sugars and starch in week 7, the leaf adjacent to the leaf used in the short-term *T*–response curve measurements was sampled. The sampled leaves were oven-dried for a minimum of 2 d at 70 °C, then ground in a ball mill and analysed for soluble sugars and starch, as described in Loveys *et al.* (2003).

To assess the likely amount of carbohydrates respired during each run of a *T*–response curve, the total amount of CO_2_ respired during each *T*–response curve was calculated (mol C m^–2^); thereafter, these values were converted to the equivalent mass of carbohydrate respired during each *T*–response curve (g m^–2^), assuming 1mol C equals 30g of carbohydrate.

### Statistical analyses

Statistical analyses were conducted in IBM SPSS^©^ Statistics for Windows, Rel. 19.0.0.2010 (SPSS Inc., Chicago, IL, USA). First, seedlings of a given drought treatment (*n*=3 for drought and *n*=3 for well-watered) were averaged within each chamber. Then, a repeated-measures analysis of variance (ANOVA) was conducted to assess differences between [CO_2_] and/or warming treatments (*n*=3) on weekly measurements of *A*_sat_ and *R*_dark_. Kolmogorov–Smirnov and Levene’s test were used to test for normality and homogeneity of variance; variables usually were log- or arcsine-transformed where necessary to meet the normality and homogeneity of variance assumptions at given time points. Over the 7 week experimental period, three-way ANOVAs were used to assess main effects of growth *T* and atmospheric [CO_2_] at given time points. When three-way ANOVAs showed no significant effect of two of the three parameters studied (e.g. growth T and [CO_2_]), independent *t*tests were used on the third parameter to determine whether there were significant differences between treatment and control plants at any given time points. Similarly, independent *t*-tests were used to assess whether water availability affected *Q*_10_ values at any given measurement *T* (within each growth *T*–atmospheric [CO_2_] treatment combination). Differences between means were considered significant at *P*<0.05.

## Results

### Establishment of the drought phenotype

**Fig. 1.**
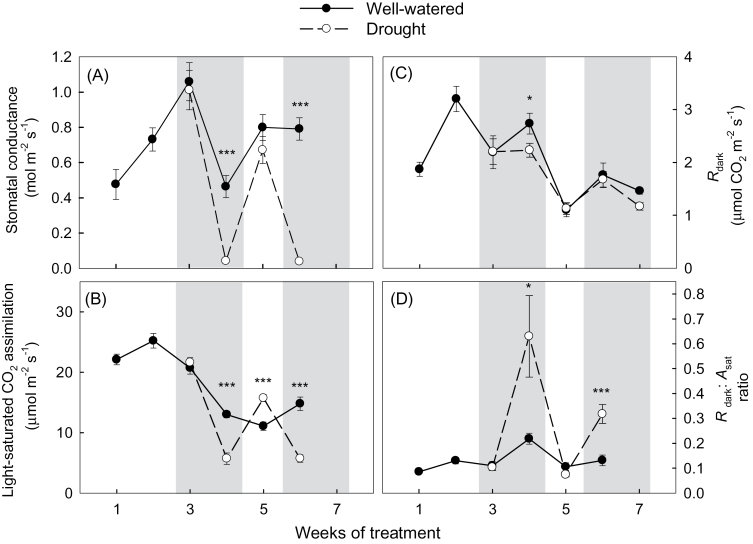
Effect of periodic drought on: (A) stomatal conductance under saturating irradiance (*g*_s_); (B) light-saturated photosynthesis (*A*_sat_); (C) leaf dark respiration (*R*_dark_); and (D) *R*_dark_/*A*_sat_ ratios of *Eucalyptus globulus* fully expanded leaves (measured over several weeks commencing early November 2010). Within each plot, values of well-watered (filled circles) and drought-treated (open circles) plants are shown (*n*=12; ±SE); values shown are averages of plants grown under two atmospheric CO_2_ concentrations (400 μmol mol^–1^ and 640 μmol mol^–1^ for ambient and elevated [CO_2_], respectively) and two growth temperatures (*T*, ambient and ambient +3°C) scenarios. The shaded regions designate two periods of controlled drought, with the intervening non-shaded region indicating when drought-treated plants were rewatered after the first drought period. Significant Student’s *t*-test *P-*values of comparisons between drought and well-watered values are indicated with * for *P*<0.1, ** for *P*<0.05, and *** for *P*<0.01. See Supplementary Figs S2–S5 at *JXB* online for plots of each parameter showing values within each [CO_2_] and growth *T* combination. Note: in week 7, a methodological error occurred when measuring gas exchange under light saturation. As such, data presented for week 7 are limited to *R*_dark_.

Drought status of the drought-treated plants was assessed via measurements of *A*_sat_, *g*_s_, and *C*_i_ each week, measured at the prevailing leaf temperature (*T*) in the mid-morning to early afternoon. In well-watered plants, *g*_s_ and *A*_sat_ exhibited week-to-week variations, reflecting concomitant variations in prevailing air/leaf *T* and vapour pressure deficit (see Supplementary Fig. S1 at *JXB* online for week-to-week fluctuations of average leaf *T*). Developmental changes in photosynthetic capacity may have also contributed to declines in *A*_sat_ (e.g. in the well-watered plants in weeks 5–6). Three-way ANOVAs showed no significant main effects of growth *T* or [CO_2_] on *A*_sat_ and *g*_s_ during the first and second drought periods (Supplementary Table S1). Hence, average *A*_sat_ and *g*_s_ values were calculated across all four [CO_2_]–*T* treatments to illustrate the overall impact of drought (Fig. 1). Similar trends in *A*_sat_ and *g*_s_ in each [CO_2_]–*T* treatment combination are shown in Supplementary Figs S2 and S4; *C*_i_ values also exhibited a similar trend, declining during each drought event (Supplementary Fig. S3). Over time, rates of *A*_sat_ in well-watered plants declined, reaching minimum values of near 10 μmol CO_2_ m^–2^ s^–1^ in week 5, with rates of *A*_sat_ increasing slightly in week 6 (Fig. 1).

Imposition of drought during the first drought period resulted in marked reductions in *A*_sat_ (compared with well-watered plants in week 4) in all [CO_2_]–*T* treatment combinations. Underpinning the drought-induced decreases in *A*_sat_ in week 4 were declines in *g*_s_ and *C*_i_ in each [CO_2_]–*T* treatment combination (Supplementary Figs S2, S3 at *JXB* online). Thereafter, *A*_sat_ of drought-treated plants recovered following rewatering in week 5 (Fig. 1; Supplementay Fig. S4). Rewatering also led to a recovery of *g*_s_ and *C*_i_ values to well-watered control plant values in all treatments, with the exception of the elevated [CO_2_] and warming treatment, where *g*_s_ values recovered to ~50% of well-watered controls (Supplementary Fig. S2). In week 6 (i.e. the first week of the second drought period), *A*_sat_ and *g*_s_ decreased significantly in drought-treated plants (*P*<0.0001; three-way ANOVA assessing main effect of H_2_O supply; Supplementary Table S1), reaching values that were similar to those at the end of the first period of drought (week 4; Fig. 1; Supplementary Figs S2, S4). Associated with the drought-mediated declines in *g*_s_ were concomitant declines in *C*_i_ (Supplementary Fig. S3). Although there were no significant interactions between growth *T*, [CO_2_], and/or H_2_O supply (in the three-way ANOVA of week 6 data), cessation of water supply clearly resulted in a rapid response to water stress in the second period of drought. In contrast to the significant effect of drought in week 6, growth *T* and [CO_2_] treatments had no significant effect on *A*_sat_ and *g*_s_ measured in week 6.

### Impact of drought, growth *T*, and elevated [CO_2_] on leaf structure and carbohydrates

Sampled leaf dry mass per unit area (LMA) did not vary significantly among the growth treatments (Tables 1, 2); a cross all treatments, the average LMA was 80.0±4.0g m^–2^. Soluble sugar concentrations varied between 5.1g m^–2^ and 9.4g m^–2^, being significantly lower (*P*=0.016) in plants grown under the elevated growth *T* treatment (compared with the ambient *T*-grown plants; Tables 1, 2). Neither atmospheric [CO_2_] nor water availability affected the concentration of soluble sugars (Tables 1, 2). Starch concentrations exhibited values ranging from 0.9g m^–2^ to 12.5g m^–2^, with neither atmospheric [CO_2_] nor growth *T* having a significant effect; in contrast, less starch was found in leaf exposed to drought (Tables 1, 2). The absence of significant treatment interaction terms for sugars and starch (Table 2) suggests that the observed effect of growth *T* and drought on sugars and starch, respectively, was consistent across the different treatments.

**Table 1.** Effect of atmospheric CO_2_ treatment (400 ppm and 640 ppm), growth temperature (T) (ambient and +3 °C), and water treatments [well-watered (WW) and drought-treated (DR)] on leaf mass per unit leaf area (LMA), soluble sugars (sucrose, glucose. and fructose), and starch (±SE, n=3)The carbohydrate equivalent needed to supply all CO_2_ respired during each run of a *T*–response curve (Fig. 2) is also shown. Also shown are: activation energy (*E*_a_) values calculated over two measuring *T* intervals below *T*_max_; for well-watered plants where *T*_max_ values were ~52 °C, the two intervals were in the 30–40 °C (low range) and 40–50°C (high range) ranges; for drought-treated plants where *T*_max_ values were ~60 °C, the intervals were in the 40–50 °C (low range) and 50–60 °C (high range) ranges. The ratio of these two *E*_a_ values is also shown.

CO_2_ treatment	Growth *T* treatment	H_2_O treatment	LMA (g m^–2^)	Sugars (g m^–2^)	Starch (g m^–2^)	Respired carbohydrate (g m^–2^)	*E*_a_–low *T* (kJ mol^–1^)	*E*_a_–high *T* (kJ mol^–1^)	*E*_a_–high T/*E*_a_–low *T* (ratio)
400 ppm	Amb	WW	75.8±14.0	7.2±1.5	9.8±1.4	0.14±0.03	41.45±1.39	30.28±3.35	0.73±0.05
		DR	95.7±22.6	9.0±3.1	3.1±1.8	0.25±0.06	35.70±8.51	43.86±12.48	1.19±0.12
	+3 °C	WW	71.5±5.4	5.4±0.4	10.0±0.7	0.14±0.01	30.21±6.32	21.20±4.98	0.78±0.22
		DR	66.0±4.7	5.1±0.6	0.9±0.3	0.20±0.02	28.26±7.25	47.39±7.03	1.76±0.17
640 ppm	Amb	WW	86.1±6.1	8.4±0.4	12.5±0.6	0.16±0.02	41.68±4.32	25.63±0.77	0.63±0.07
		DR	91.0±12.0	9.4±1.9	6.4±3.0	0.19±0.05	49.50±3.11	39.27±2.52	0.80±0.06
	+3 °C	WW	75.1±3.2	5.5±0.1	11.6±1.0	0.13±0.02	39.37±4.76	33.21±8.46	0.93±0.37
		DR	82.1±12.5	7.5±1.9	4.1±1.8	0.16±0.01	34.55±0.52	26.92±9.90	0.77±0.28

See Table 2 for results of three-way ANOVAs of selected traits.

**Table 2.** Three-way ANOVAs of leaf mass per unit leaf area (LMA), area-based concentrations of soluble sugars and starch, and the temperature where *R*_dark_ reached its maximum (*T*_max_)Main factors used in the analysis were atmospheric growth CO_2_ concentration (CO_2_), growth temperature (*T*), and water availability (H_2_O).

Source (growth environment)	df	LMA	Sugars	Starch	*T*_max_	*E*_a_– low *T*	*E*_a_– high *T*	Ratio
CO_2_	1	0.319	0.213	0.106	0.962	0.063	0.399	**0.031**
*T*	1	0.159	**0.016**	0.370	0.568	0.**027**	0.620	0.126
H_2_O	1	0.538	0.420	**<0.001**	**<0.001**	0.755	**0.032**	**0.019**
CO_2_×*T*	1	0.802	0.939	0.804	0.684	0.924	0.970	0.544
CO_2_×H_2_O	1	0.961	0.656	0.275	0.824	0.479	0.132	**0.021**
*T*×H_2_O	1	0.509	0.959	0.407	0.324	0.558	0.725	0.734
CO_2_×*T*×H_2_O	1	0.434	0.466	0.682	0.792	0.282	0.131	0.149
Error	16							

*P*-values in bold indicate significant effects (*P*<0.05). See Table 1 for trait values of LMA, sugars, and starch; see Fig. 3 for *T*_max_ values.

### Impact of drought, growth *T*, and elevated [CO_2_] on leaf respiration

Rates of *R*_dark_ (measured at the prevailing mid-morning to early afternoon temperature) varied with time (Fig. 1), reflecting, in part, week-to-week variations of prevailing leaf *T* (Supplementary Fig. S1 at *JXB* online). Importantly, the effect of the treatment combinations on *R*_dark_ (Fig. 1; Supplementary Fig. S5) was considerably less than the drought-mediated changes in *A*_sat_ (Fig. 1; Supplementary Fig. S4). Indeed, a three-way ANOVA showed no significant main effects of growth *T*, [CO_2_], and/or drought *R*_dark_ in week 6 (Fig. 1; Supplementary Fig. S5). Thus, in contrast to *A*_sat_ and *g*_s_ (Fig. 1; Supplementary Figs S2, S4), none of the growth treatments (including drought) had a significant effect on rates of leaf *R*_dark_ measured at the prevailing *T* occurring at the time of mid-morning/early afternoon measurements. Consequently, *R*_dark_:*A*_sat_ ratios were consistently higher in drought-treated plants (both drought periods) in all treatments, with rewatering after the first drought period (week 5) resulting in a sharp decline in *R*_dark_:*A*_sat_ in all growth *T* and [CO_2_] treatments (Fig. 1; Supplementary Fig. S6). In week 6, a three-way ANOVA (Supplementary Table S1) revealed a significant main effect of drought on the *R*_dark_:*A*_sat_ ratio (*P*<0.001). Thus, drought altered the instantaneous carbon balance of *E. globulus* leaves in the experiment, irrespective of the growth [CO_2_] and/or growth *T* treatment.

### Drought-mediated changes in the temperature response of dark respiration

Drought had little effect on rates of *R*_dark_ measured at the prevailing mid-morning/early afternoon *T* of each treatment combination (Fig. 1; Supplementary Fig. S5 at *JXB* online). Typically, these *T* values were in the 18–35 °C range (with some leaf *T* values reaching 38 °C). However, it remained unclear whether the treatments affected the shape of the short-term *T*–response curves of leaf *R*_dark_ over a wider temperature range than 18–35 °C (including *T* exceeding *T*_max_).

**Fig. 2.**
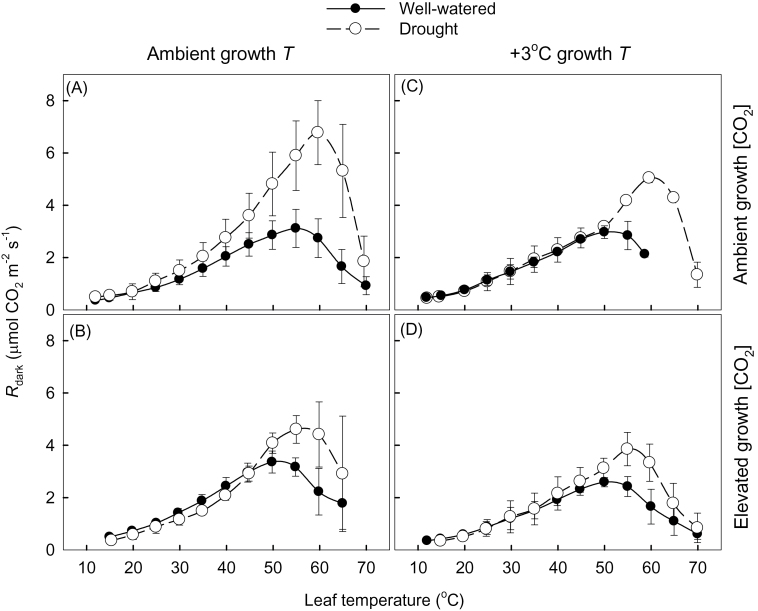
Effect of drought on short-term temperature (*T*)–response curves of area-based leaf respiration measured in darkness (*R*_dark_) of *Eucalyptus globulus* fully expanded leaves for plants grown under two atmospheric CO_2_ concentrations (400 μmol mol^–1^ and 640 μmol mol^–1^ for ambient and elevated [CO_2_], respectively) and two growth *T* (ambient and ambient +3°C) scenarios: (A) ambient [CO_2_] and ambient *T*; (B) elevated [CO_2_] and ambient *T*; (C) ambient [CO_2_] and elevated *T*; and (D) elevated [CO_2_] and elevated *T*. Within each [CO_2_]–growth T combination, values are shown for well-watered (filled symbols) and drought-treated (open symbols) plants. Measurements took place in week 7 when drought-treated leaves were in the second week of the second period of drought. Leaves were heated at a rate of 1 °C min^–1^, starting at 10–15 °C; data were recorded at minute intervals. To allow comparison of treatments at designated leaf *T*, only values at 5 °C intervals are shown, using the nearest *R*_dark_ and leaf *T* values to each 5 °C value. Values shown are the mean of three replicates (±SE).

Different treatment combinations affected the shape of the *R*_dark_–*T* curves (Fig. 2; shown at 5 °C intervals to simplify the presentation). Over the 15–45 °C range, there was little difference in rates of *R*_dark_ between well-watered and drought-treated plants (Fig. 2), consistent with treatment effects on *R*_dark_ measured at the prevailing temperatures. Moreover, a three-way ANOVA revealed that at leaf *T* of 15, 25, and 45 °C, there were no significant differences among any of the three treatments (growth *T*, growth [CO_2_], and/or drought). In contrast, marked differences between well-watered and drought-treated plants were observed at leaf *T* values >45 °C, with drought-treated plants exhibiting markedly higher *R*_dark_ at *T*_max_ (i.e. *R*_max_) than their well-watered counterparts (Fig. 2). Thus, while growth *T*, growth [CO_2_], and water availability had little impact on *R*_dark_ at *T* <45 °C, drought increased *R*_dark_ at *T* >45 °C.

**Fig. 3.**
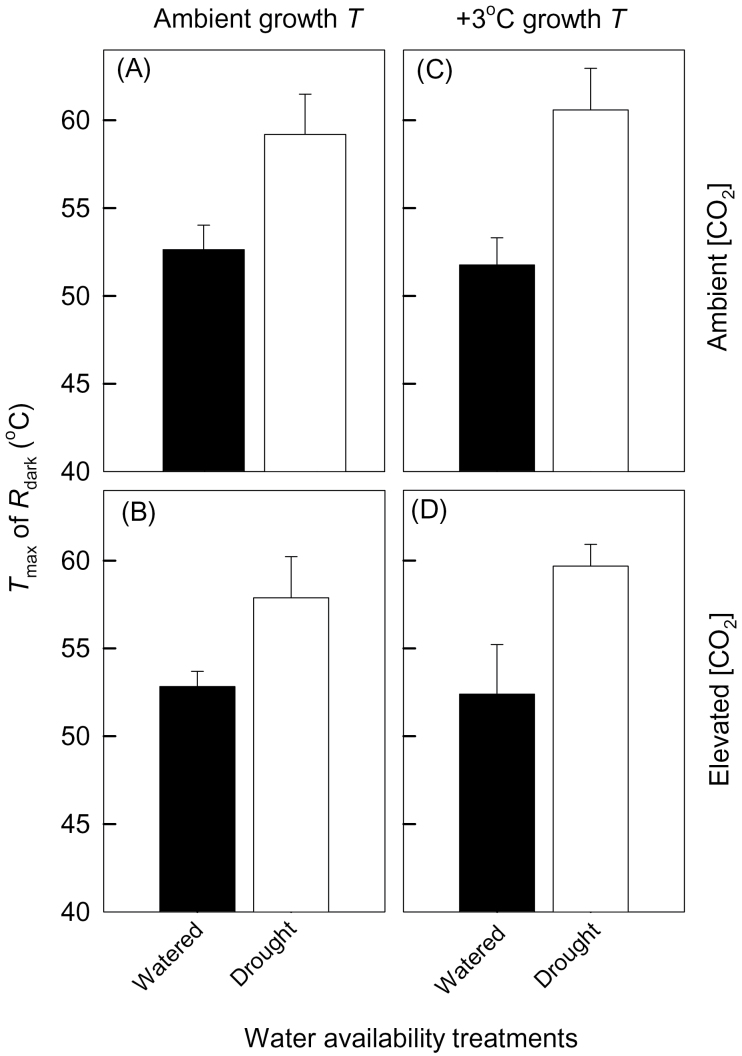
Temperature where *R*_dark_ reached its maximum (*T*_max_) of *Eucalyptus globulus* fully expanded leaves for plants grown under two atmospheric CO_2_ concentarions (400 μmol mol^–1^ and 640 μmol mol^–1^ for ambient and elevated [CO_2_], respectively) and two growth *T* (ambient and ambient +3 °C) scenarios: (A) ambient [CO_2_] and ambient *T*; (B) elevated [CO_2_] and ambient *T*; (C) ambient [CO_2_] and elevated *T*; and (D) elevated [CO_2_] and elevated *T*. Within each [CO_2_]–growth T combination, values are shown for well-watered (filled bars) and drought-treated (open bars) plants. Measurements took place in week 7 when drought-treated leaves were in the second week of the second period of drought. Data shown were derived from short-term *T*–response curves of mass-based *R*_dark_ (Fig. 5). Values shown are the mean of three replicates (±SE).

Drought increased *T*_max_ such that well-watered and drought-treated plants exhibited *T*_max_ values of 52.4±0.5 °C and 59.8±1.5 °C, respectively (when averaged across both growth [CO_2_] and growth *T* treatments; Fig. 3). Importantly, only drought had a significant effect on *T*_max_ (*P*<0.0001), with no main or interactive effects of growth *T* and atmospheric [CO_2_] on *T*_max_ (Table 2). Taken together, these results indicate that drought affected the shape of the temperature–response curve of *R*_dark_, especially at high temperatures (>45 °C) in leaves of young *E. globulus* trees, whereas growth *T* and elevated CO_2_ had no significant effect on *R*_dark_ at *T*_max_ or the value *T*_max_ *per se* (Fig. 3).

**Fig. 4.**
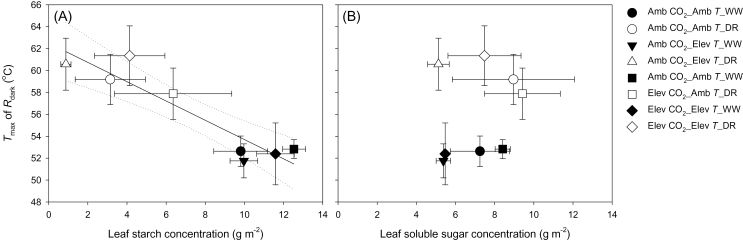
Relationship between temperature where *R*_dark_ reached its maximum (*T*_max_) and (A) leaf starch and (B) soluble sugar concentrations (g m^–2^) of *Eucalyptus globulus* with values of *T*_max_ taken from Fig. 3 and starch/sugar concentrations from Table 1. Values shown are the mean of three replicates (±SE). Analyses of starch and sugar were carried out on a different set of (unheated) leaves from those used for the *R*_dark_–*T* measurements. In (A), linear regression revealed a significant negative relationship (*P*<0.001; *r* ^2^=0.82) between *T*_max_ and starch concentration (regression shown with a solid line, with dotted lines showing 95% confidence intervals). In contrast, there was no significant relationship between *T*_max_ and concentration of soluble sugars (B). See Table 1 for treatment means of sugar and starch concentrations.

Previous studies have linked variations in high temperature tolerance of photosynthesis and respiration to variations in soluble sugar concentrations (Seemann *et al.*, 1986; Hüve *et al.*, 2006, 2012). Given this, the relationship between *T*_max_ (Fig. 3) and the concentration of soluble sugars (Table 1) was analysed using linear regression. No relationship was found (*P*=0.562, *r* ^2^=0.06) (Fig. 4B). However, a highly significant relationship was found between *T*_max_ and starch concentrations (*P*<0.001, *r* ^2^=0.84), with increased starch in well-watered plants associated with a decrease in *T*_max_ (*T*_max_=62.51–0.88×[starch]; Fig. 4A) compared with drought-treated plants.

To gain insights into the percentage of leaf sugar and starch that may have been respired during each *T*–response curve, the total amount of CO_2_ respired during each *T*–response curve (mol C m^–2^) was calculated; thereafter, these values were converted to an equivalent mass of carbohydrate respired during each *T*–response curve. The total carbohydrate respired ranged from 0.13g m^–2^ to 0.25g m^–2^ (Table 1), representing <4% of total soluble sugars present in non-heated leaves. Therefore, for all treatments, *R*_dark_ during the *T*–response curve runs is unlikely to have been limited by substrate availability. Moreover, starch degradation [which has been linked to abrupt increases in respiration rates at very high leaf *T* values (Hüve *et al.*, 2012)] is likely to have continued during the entire *T*–response curve runs in all treatments, as the total carbohydrate respired represented only 1–23% of leaf starch (Table 1).

### Impact of drought, growth *T*, and elevated [CO_2_] on the *Q*_10_ and respiratory ‘burst’

**Fig. 5.**
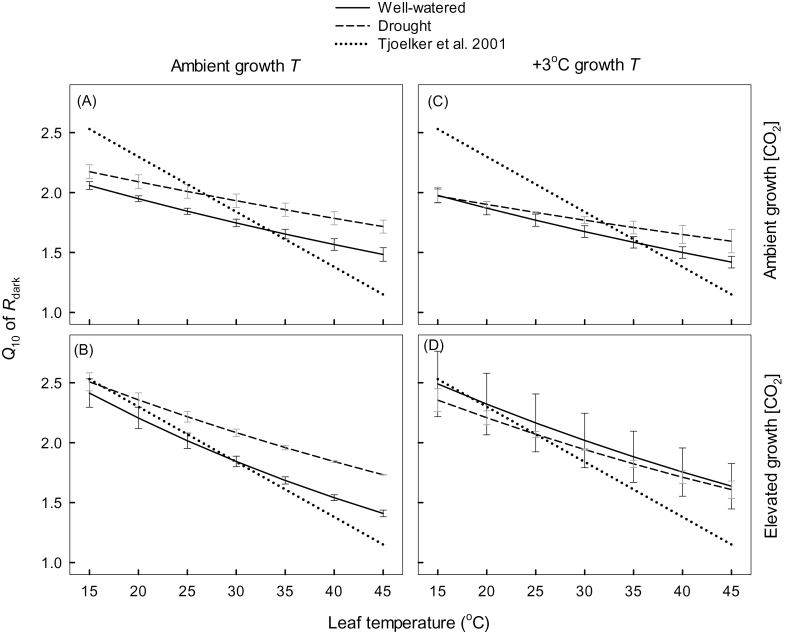
Effect of drought on *Q*_10_ of *R*_dark_ [i.e. proportional increase in leaf *R*_dark_ per 10 °C increase in leaf temperature (*T*)] at 5 °C *T* intervals for *Eucalyptus globulus* fully expanded leaves for plants grown under two atmospheric CO_2_ concentrations (400 μmol mol^–1^ and 640 μmol mol^–1^ for ambient and elevated [CO_2_], respectively) and two growth *T* (ambient and ambient +3 °C) scenarios: (A) ambient [CO_2_] and ambient *T*; (B) elevated [CO_2_] and ambient *T*; (C) ambient [CO_2_] and elevated *T*; and (D) elevated [CO_2_] and elevated *T*. Within each [CO_2_]–growth *T* combination, values are shown for well-watered (solid line) and drought-treated (dashed line) plants. For comparison, the *Q*_10_–*T* relationship reported in Tjoelker *et al.* (2001) (i.e. *Q*_10_=3.22–0.046*T*). Measurements took place in week 7 when drought-treated leaves were in the second week of the second period of drought. At any measuring *T*, *Q*_10_ values were calculated using second-order polynomial curves fitted to log *R*_dark_ versus *T* over the 15–45 °C range, with *Q*_10_ values being calculated from the slope at any *T* (using equations 1–3 in the main text). See Table 1 for a three-way ANOVA comparing treatment effects at any set measuring *T*.

To assess treatment effects on *T* sensitivity, individual plant log-transformed rates of *R*_dark_ were first plotted against *T*; thereafter, second-order polynomial equations were fitted to the log-*R*_dark_–*T* plots over the 15–45 °C range, with the slope of those curve fits then used to calculate the *Q*_10_ of *R*_dark_ at any measuring *T* (see equations 1–3). Figure 5 shows the resulting *Q*_10_–*T* plots for each treatment combination, comparing well-watered and drought-treated plants within each growth *T*–[CO_2_] combination. In each panel, the *Q*_10_–*T* relationship reported by Tjoelker *et al.* (2001) is shown for comparison (*Q*_10_=3.05–0.045*T*). Regardless of the growth treatment combination, the observed *Q*_10_ values were consistently higher at leaf *T* >35 °C than those reported by Tjoelker *et al.* (2001). Moreover, three-way ANOVAs conducted at individual measuring *T* (at 35, 40, and 45 °C) revealed a significant effect of water supply on *Q*_10_ values (Table 3), with *Q*_10_ values being higher in drought compared with well-watered plants (Fig. 5). In contrast, growth *T* and [CO_2_] had no significant effect on the *T* sensitivity of *R*_dark_ at high measurement *T* (Table 3). However, when comparisons of *Q*_10_ values were made at lower leaf *T* (<35 °C), significant differences were found between the two growth [CO_2_] (Table 3), with *Q*_10_ values being higher in plants grown under elevated [CO_2_] than in those grown under ambient [CO_2_]. Taken together, these results demonstrate that: (i) the Tjoelker *et al.* (2001) relationship consistently underestimates the *T* sensitivity of leaf *R*_dark_ of *E. globulus* at high leaf *T* (>35 °C); (ii) the effectiveness of the Tjoelker *et al.* (2001) equation in predicting *Q*_10_ values at low measuring *T* differs between plants grown under ambient and elevated [CO_2_] (being better under elevated [CO_2_] conditions); and (iii) drought increases the *T* sensitivity of *R*_dark_ especially at high measurement *T*.

**Table 3.** Three-way ANOVAs of *Q*_10_ values of leaf *R*_dark_ at designated measuring temperatures in week 7 (i.e. second drought period)*Q*_10_ values (see Fig. 5) were calculated using second-order polynomial curves fitted to log *R*_dark_ versus *T* over the over the 15–45 °C range, with *Q*_10_ values being calculated from the slope at any set measuring *T* (using equations 1–3 in the main text). Main factors used in the analysis were atmospheric growth CO_2_ concentration (CO_2_), growth temperature (*T*), and water availability (H_2_O).

Source (growth environment)	df	Instantaneous measurement *T*						
		7	20	25	30	35	40	45
CO_2_	1	**0.000**	**0.001**	**0.002**	**0.007**	**0.038**	0.165	0.493
*T*	1	0.302	0.326	0.369	0.434	0.521	0.631	0.736
H_2_O	1	0.836	0.488	0.230	0.093	**0.039**	**0.019**	**0.011**
CO_2_×*T*	1	0.548	0.444	0.358	0.297	0.264	0.251	0.256
CO_2_×H_2_O	1	0.653	0.652	0.649	0.647	0.643	0.645	0.648
*T*×H_2_O	1	0.318	0.223	0.159	0.122	0.106	0.104	0.115
CO_2_×*T*× H_2_O	1	0.753	0.607	0.479	0.375	0.307	0.269	0.249
Error	16							

*P*-values in bold indicate significant effects (*P*<0.05).

To assess whether a ‘respiratory burst’ occurred in each treatment, *E*_a_ values were calculated over two *T* intervals below the *T*_max_. For well-watered plants, *E*_a_ values were calculated within two ranges of leaf *T*: within the 30–40 °C (low-*T* range) and within the 40–50 °C (high-*T* range) ranges; for drought-treated plants, the equivalent ranges used were 40–50 °C and 50–60 °C. For well-watered plants, *E*_a_ values over the high-*T* range were lower compared with their low-*T* counterparts; the same was true for drought-treated plants grown under elevated growth [CO_2_] (Table 1). In contrast, *E*_a_ values over the high-*T* range were greater than those over the corresponding low-*T* range in drought-treated plants grown under ambient [CO_2_] (Table 1). Indeed, high-*T*/low-*T* ratios were significantly affected by growth [CO_2_] and water availability; there was also a significant growth [CO_2_]×water interaction (Table 2), suggesting that the extent to which drought affected the respiratory burst did indeed differ between the two growth [CO_2_] treatments. There was no evidence of growth *T* affecting the presence/absence of a respiratory burst. Taken together, these observations suggest that while respiratory bursts were minor or absent in non-stressed *E. globulus* seedlings, more pronounced bursts occurred in ambient [CO_2_]-grown plants subjected to drought.

## Discussion

The present study sought to assess the effects of three climate change drivers (elevated growth [CO_2_], elevated growth *T*, and drought) on the short-term *T* sensitivity of leaf *R*_dark_ of *E. globulus*. Although no significant effects of elevated [CO_2_] and growth temperature on *R*_dark_ were observed, the results highlight the importance of drought in: (i) increasing rates of *R*_dark_ at high leaf *T* typical of heatwave events; and (ii) increasing the *T* at which maximal rates of *R*_dark_ occur (*T*_max_). It was also found that increased [CO_2_]: (i) increased the slope of *Q*_10_–*T* relationships of respiration, particularly at low to moderate measuring *T*; and (ii) determined the extent to which the respiratory burst increases under drought. Collectively, the study highlights the dynamic nature of the *T* dependence of *R*_dark_ in plants experiencing future climate change scenarios, particularly with respect to drought and elevated [CO_2_]. Importantly, the lack of interactive effects among the three treatments (elevated [CO_2_], growth *T*, and drought) suggests that the effect of drought on rates of *R*_dark_ might not be altered in the near future by increases in atmospheric [CO_2_] or growth *T*. The response to more extreme climate change scenarios is, however, not known.

### Drought impacts on respiration rates

The hypothesis that *R*_dark_ at moderate to high leaf *T* would be inhibited by drought (particularly under elevated growth *T* and ambient atmospheric [CO_2_]) was based on past work showing that leaf *R*_dark_ can be substrate limited (Azcón-Bieto and Osmond, 1983), particularly at high measuring *T* (Atkin and Tjoelker, 2003; Bunce, 2007), and because, in many cases, drought reduces leaf *R*_dark_ at a set measuring *T* (Flexas *et al.*, 2005; Atkin and Macherel, 2009; Crous *et al.*, 2011; Rodríguez-Calcerrada *et al.*, 2011; Duan *et al.*, 2013). When measured at low to moderate *T* (15–35 °C), it was found that drought did not inhibit leaf *R*_dark_ in the growth *T* and/or [CO_2_] treatments (Supplementary Fig. S5 at *JXB* online). On first inspection, this result appears to contradict the assertion that drought inhibits leaf *R*_dark_ (Flexas *et al.*, 2005; Atkin and Macherel, 2009). However, closer inspection of past studies reveals that in approximately one-third of cases, drought does not affect *R*_dark_ at ≤25 °C (Galmés *et al.*, 2007; Gimeno *et al.*, 2010). Similarly, the resilience of *R*_dark_ in drought-treated plants was observed over the low to moderate range of *T*. Despite marked differences in total non-structural carbohydrates between well-watered and drought-treated plants, leaf *R*_dark_ remained unchanged over the 15–35 °C range, suggesting that respiratory metabolism was not substrate limited across this lower *T* range. Indeed, the analysis of the total amount of carbon respired during each run (0.13–0.25g m^–2^) was <4% of the sugar present in leaves prior to measurements (Table 1), suggesting that for all treatments, *R*_dark_ was unlikely to have been substrate limited across all measurement *T* values.

When measured at leaf *T* below 35 °C, *Q*_10_ values were higher in plants grown under elevated [CO_2_] than in those grown under ambient [CO_2_]. Why was this? Higher *Q*_10_ values have been linked to metabolic conditions where respiratory flux is more limited by enzymatic capacity than when *R*_dark_ is limited by substrate supply and/or turnover of ATP to ADP (Atkin and Tjoelker, 2003). Given this, one possibility is that growth under elevated [CO_2_] alters which factors limit respiratory flux over the low to moderate *T* range (i.e. away from substrate/ATP turnover to enzyme capacity). Further work that quantifies *in vivo* limitations in each of these factors is needed.

Interestingly, it was found that drought increased *R*_dark_ at high measuring leaf *T* >35 °C (Fig. 2), resulting in significant increases in *R*_max_ and *T*_max_ (Figs 2, 3) and increased *Q*_10_ of leaf *R*_dark_ at leaf *T* >35 °C (Fig. 4; Table 1). Past reports have reported drought-mediated increases in leaf *R*_dark_ at moderate measuring *T* (Zagdanska, 1995; Bartoli *et al.*, 2005; Slot *et al.*, 2008; Metcalfe *et al.*, 2010) and drought-mediated increases in the *Q*_10_ (Slot *et al.*, 2008) in a small number of species. Thus, while the results differ somewhat from these studies, it is apparent that when leaves are exposed to high *T* (>40 °C), drought may exacerbate/increase *R*_dark_ rates (Slot *et al.*, 2008; Metcalfe *et al.*, 2010; Flanagan and Syed, 2011). While the underlying mechanisms responsible for these higher rates of leaf *R*_dark_ and *Q*_10_ values at high leaf *T* (in drought-treated plants) remain unclear, it seems unlikely that this response was linked to substrate supply differences (see above). Given this, it is suggested that drought-mediated increases in the demand for respiratory products (e.g. ATP and/or NADH) by cellular maintenance processes [e.g. high rates of protein turnover and maintenance of ion gradients (Amthor, 2000; Scheurwater *et al.*, 2000) and membrane stability] may have played a role in the increased rates of leaf *R*_dark_ at very high *T*.

### Coupling of respiratory and photosynthetic metabolism

Past studies have reported that variation in *R*_dark_ is often tightly coupled to variation in *A*_sat_ (Gifford, 1995; Loveys *et al.*, 2003; Whitehead *et al.*, 2004; Noguchi and Yoshida, 2008). Given that neither growth *T* nor elevated [CO_2_] resulted in significant changes in *A*_sat_ in the juvenile leaves of *E. globulus* (Fig. 1), the absence of growth *T* and/or [CO_2_] effect on *R*_dark_ is perhaps not surprising. In the case of elevated [CO_2_], other studies have also reported no effect of elevated [CO_2_] on rates of leaf *R*_dark_ (Tissue *et al.*, 2002; Bunce, 2005). Less common is the absence of a significant growth *T* effect on rates of *R*_dark_, as respiration more often than not acclimates to sustained changes in growth *T* (Atkin *et al.*, 2000*a*; Bolstad *et al.*, 2003; Loveys *et al.*, 2003; Tjoelker *et al.*, 2008, 2009; Zaragoza-Castells *et al.*, 2008). However, given that the plants in the present study experienced weekly temperature changes of ~5–10 °C due to springtime weather at that time (Supplementary Fig. S1 at *JXB* online), it was not expected to see a growth *T* effect of +3 °C warming on rates of *R*_dark_. Hence, this changing weather pattern did not accommodate the potential acclimation of *R*_dark_ to sustained changes in growth temperature.

Although rates of *R*_dark_ (<35 °C) were relatively unaffected by any of the treatments, drought did have a marked inhibitory effect on *A*_sat_. As such, *R*_dark_:*A*_sat_ ratios increased markedly under drought (Fig. 1; Supplementay Fig. S6 at *JXB* online). This finding is similar to that of a recent study assessing the effect of drought on *Eucalyptus saligna* saplings, *R*_dark_:*A*_sat_ increased 56% under drought (reflecting the greater inhibitory effect of drought on *A*_sat_ than on *R*_dark_) (Ayub *et al.*, 2011). Similarly, *R*_dark_:*A*_sat_ ratios have been found to increase markedly under drought in evergreen and deciduous Mediterranean forests (Zaragoza-Castells *et al.*, 2008; Rodríguez-Calcerrada *et al.*, 2010). Variations in *R*_dark_:*A*_sat_ ratios have also been reported in plants acclimated to contrasting growth *T* and [CO_2_] in previous studies (Campbell *et al.*, 2007; Tingey *et al.*, 2007; Ow *et al.*, 2008; Cai *et al.*, 2010; Ayub *et al.*, 2011). In contrast, no significant effect of elevated growth [CO_2_] or elevated growth *T* on the ratio between respiration and photosynthesis was found (i.e. *R*_dark_:*A*_sat_ ratios were homeostatic), reflecting the lack of [CO_2_] or growth *T* effects on C gain and C loss. Thus, these observations highlight the variable responses of *R*_dark_:*A*_sat_ ratios to climate change factors, and the need for predictive dynamic vegetation–climate models to exercise caution when assuming a constant *R*_dark_:*A*_sat_ ratios.

### Temperature response curves under future climate change scenarios

Unlike past studies assessing the effect of climate change drivers on the *T* response of *R*_dark_ that relied on low resolution data collected over a narrow *T* range, here high resolution curves were generated over a wide *T* range, including lethally high *T* where respiratory function was inhibited. The experiments revealed several marked effects of drought and elevated [CO_2_] on the shape of the resultant *R*_dark_–*T* curves. At *T* <35 °C, little treatment (drought and CO_2_) difference could be detected in specific rates of *R*_dark_ at any *T* (Fig. 2)—yet, analysis of the *Q*_10_ values over the 15–35 °C range revealed significantly higher *Q*_10_ values in elevated [CO_2_] compared with plants grown under ambient [CO_2_] (Fig. 5; Table 1). This finding was consistent across both growth *T*, suggesting that *R*_dark_ may be more *T* sensitive (at *T* <35 °C) in a future, higher [CO_2_] world, at least in juvenile leaves of fast-growing trees. Above 35 °C, other factors (e.g. drought) appear to play a more important role in determining the *Q*_10_ response. Given the importance of the *T* dependence of *R*_dark_ for carbon storage by terrestrial ecosystems (Huntingford *et al.*, 2013; Wythers *et al.*, 2013), such changes in the *T* dependence of *R*_dark_ have the potential to alter the potential of managed forest ecosystems to sequester atmospheric CO_2_ markedly. However, given that the present work was limited to seedlings, further work is needed to assess clearly whether the same responses occur in mature trees of managed and natural forests.

One of the most striking outcomes of this study was the effect drought had on the shape of *R*_dark_–*T* curves when *T* exceeded 45 °C. Drought-treated plants exhibited a 7 °C increase in *T*_max_ (59.8±1.5 °C) compared with well-watered plants (52.4±0.5 °C) when averaged across all growth [CO_2_] and growth *T* treatments. In the survey of the literature by Tjoelker *et al.* (2001) (that relied on curve fits to low resolution *R*–*T* data for measuring *T* <35°C), it was predicted that a globally averaged *T*_max_ value was likely to be near 48 °C. Given that actual *T*_max_ values were not available in the data reported by Tjoelker *et al.* (2001), it seems unlikely that *T*_max_ (i.e. the *T* where *Q*_10_=1.0) can be accurately predicted from curve fits to *R–T* data over a sublethal range of *T*. In the present study, *T*_max_ values predicted via extrapolation from curve fits over the 15–45 °C range were 59–69 °C for well-watered plants, and 76–94 °C for drought-treated plants (data not shown). Yet, actual measured *T*_max_ values were markedly lower, being 52–53 °C for well-watered and 58–61 °C for drought-treated plants (Fig. 3). Moreover, while past studies on *Populus tremula* or *Quercus* sp. showed that actual values of *T*_max_ were near 48–50 °C for these species (Hamerlynck and Knapp, 1994; Hüve *et al.*, 2012), O’Sullivan *et al.* (2013) found that the *T*_max_ of *E. pauciflora* trees growing in several thermally contrasting environments ranged from 51 °C to 57 °C (i.e. markedly greater than 48 °C). From these observations, it is suggested that extrapolated curves fitted to *R*–*T* plots over a range of sublethal *T* values do not necessarily provide an accurate prediction of actual *T*_max_ values.

While the *T*_max_ of *R*_dark_ is not a measure of thermotolerance (i.e. the ability of metabolic processes to withstand high *T*), variations in *T*_max_ are positively correlated with variations in the temperature where disruption of electron transport in photosystem II occurs (typically in the 42–55 °C range; Havaux *et al.*, 1991; Knight and Ackerly, 2002; Hüve *et al.*, 2012; O’Sullivan *et al.*, 2013). Thus, adding to past reports on drought-induced increases in photosynthetic high *T* tolerance (Seemann *et al.*, 1986; Havaux, 1992), the present study shows for the first time that drought can also increase respiratory heat tolerance. This finding has relevance given that: (i) 23% of the Earth’s land surface habitats exhibit air temperatures of >40 °C (Singsaas *et al.*, 1999; Wise *et al.*, 2004), which in turn can result in leaf *T* exceeding 50 °C (Hamerlynck and Knapp, 1994); and (ii) high leaf *T* are likely to become more common in the future based on radiative warming (Meehl and Tebaldi, 2004; IPCC, 2007; Duffy and Tebaldi, 2012). Further work is needed to assess whether the present findings are representative of a wider range of species growing in drought-suceptible biomes around the world.

Past studies have reported that increases in leaf osmotic potential and soluble sugar concentrations are associated with increases in enhanced heat tolerance of both photosynthesis and respiration, possibly via sugars increasing protection of chloroplast and mitochondrial membranes (Seemann *et al.*, 1986; Hüve *et al.*, 2006, 2012). More broadly, increased tolerance of heat stress can occur via changes in membrane fluidity that result from modifications in membrane lipid and protein composition (Björkman *et al.*, 1980; Sung *et al.*, 2003). Increased synthesis of isoprene at high *T* may also help stabilize membranes (Sharkey, 2005; Velikova *et al.*, 2011), with isoprene synthesis linked to increased use of starch and soluble sugars in some drought-stressed plants (Funk *et al.*, 2004; Monson *et al.*, 2012; Rodriguez-Calcerrada *et al.*, 2013). Given this, what might we expect from a relationship between the *T*_max_ of *R*_dark_ and concentrations of non-structural carbohydrates? Although Hüve *et al*. (2012) found that additional leaf soluble sugars (supplemented via petiole uptake) increased the *T*_max_, no relationship between initial sugar concentrations and *T*_max_ was found in the present study (Fig. 4). However, a strong negative relationship between *T*_max_ and starch concentrations was found (Fig. 4). One possible explanation was that the maintenance of soluble sugar concentrations was facilitated by starch degradation (leading to lower concentrations in drought-stressed plants; Table 1), with products of sugar metabolism providing the glycolytic molecules [e.g. phosphoenolpyruvuate (PEP)] needed for isoprene synthesis (Lichtenthaler, 1999). Alternatively, starch degradation and homeostasis of sugar pools may have provided the carbon molecules necessary for synthesis of compatible solutes [e.g. methylated cyclic amino acids (Lippert and Galinski, 1992), glycine betaine (Wani *et al.*, 2013), or trehalose (Penna, 2003)], several of which are known to increase heat tolerance in plant cells. Irrespective of the mechanism via which *T*_max_ was increased in drought-treated plants, most probably the products of starch degradation played a role.

Recently, Hüve *et al*. (2012) proposed that accelerated starch degradation might be responsible for the abrupt increase in *R*_dark_ at very high leaf *T* (i.e. ‘respiratory burst’). In their study, pulse–chase experiments strongly suggested that leaf *R*_dark_ of *P. tremula* was substrate limited at moderate and high leaf *T* values, with accelerated starch degradation at high leaf *T* probably alleviating substrate limitations of respiratory metabolism, resulting in a ‘burst’ of *R*_dark_. In the present study, little evidence of a strong respiratory ‘burst’ was found in a majority of the treatments (Table 1), despite most treatments exhibiting similar area-based starch concentrations (in leaves that were not subjected to the *T*–response curve protocol) to those in Hüve *et al*. (2012). Thus, it may be premature to attribute presence/absence of a respiratory burst in all species to accelerated rates of starch degradation at high leaf *T*.

## Conclusions

The present study has shown that predicted climate change scenarios may markedly alter the shape of *T*–response curves of *R*_dark_, particularly when considering the effect of drought on *R*_dark_ at high leaf *T* indicative of heatwave events. Yet, changes in *T* dependence of *R*_dark_ were also apparent when comparing *Q*_10_ values at low to moderate *T* of plants grown under ambient and elevated [CO_2_] (being highest under elevated [CO_2_] over the 15–35 °C range), but not in ambient and elevated temperature treatments. Interestingly, no evidence of interactive effects between atmospheric [CO_2_], growth *T*, or water availability was found, suggesting that the stimulatory effects of elevated [CO_2_] (low to moderate *T*) and drought (at high *T*) on the *Q*_10_ are generalized phenomena, at least for *E. globulus* seedlings grown under semi-controlled environment conditions. Collectively, these results challenge the prevailing assumption in most climate models that the *T* dependence of *R*_dark_ is constant (Huntingford and Cox, 2000). If more widespread, the present results suggest that dynamic changes in the shape of *R*_dark_–*T* curves may occur in the future, in response to rising levels of atmospheric [CO_2_] and increasing frequency and severity of drought. Such changes, if realized, have important implications for terrestrial C storage and atmospheric [CO_2_] in a future, warmer world.

## Supplementary data

Supplementary data are available at *JXB* online.

Figure S1. Temporal variations in leaf temperature.

Figure S2. Temporal variation in stomatal conductance.

Figure S3. Temporal variation in internal CO_2_ concentration.

Figure S4. Temporal variation in light-saturated photosynthesis (*A*_sat_).

Figure S5. Temporal variation in leaf respiration (*R*_dark_).

Figure S6. Temporal variation in *R*_dark_:*A*_sat_ ratios.

Table S1. Statistical analyses of leaf gas exchange.

Supplementary Data

## Abbreviations:

*A*_sat_: light-saturated photosynthesis
*Q*_10_: proportional change in respiration per 10 °C rise in *T*
*R*_dark_: dark respiration
T: temperature
WTC: whole tree chamber.

## References

[CIT0001] Adams HD Guardiola-Claramonte M Barron-Gafford GA Villegas JC Breshears DD Zou CB Troch PA Huxman TE 2009 Temperature sensitivity of drought-induced tree mortality portends increased regional die-off under global change-type drought. Proceedings of the National Academy of Sciences, USA 106, 7063–7066. 10.1073/pnas.0901438106 PMC2678423 19365070

[CIT0002] Amthor JS 2000 The McCree–de Wit–Penning de Vries–Thornley respiration paradigms: 30 years later. Annals of Botany 86, 1–20.

[CIT0003] Armstrong AF Badger MR Day DA Barthet MM Smith PMC Millar AH Whelan J Atkin OK 2008 Dynamic changes in the mitochondrial electron transport chain underpinning cold acclimation of leaf respiration. Plant, Cell and Environment 31, 1156–1169. 10.1111/j.1365-3040.2008.01830.x 18507806

[CIT0004] Atkin OK Atkinson LJ Fisher RA Campbell CD Zaragoza-Castells J Pitchford J Woodward FI Hurry V 2008 Using temperature-dependent changes in leaf scaling relationships to quantitatively account for thermal acclimation of respiration in a coupled global climate–vegetation model. Global Change Biology 14, 2709–2726.

[CIT0005] Atkin OK Botman B Lambers H 1996 The causes of inherently slow growth in alpine plants: an analysis based on the underlying carbon economies of alpine and lowland *Poa* species. Functional Ecology 10, 698–707.

[CIT0006] Atkin OK Bruhn D Tjoelker MG 2005 Response of plant respiration to changes in temperature: Mechanisms and consequences of variations in Q_10_ values and acclimation. In: Lambers H Ribas-Carbó M, eds. Plant respiration: from cell to ecosystem. Advances in Photosynthesis and Respiration Volume 18. Dordrecht: Springer, 95–135.

[CIT0007] Atkin OK Evans JR Ball MC Lambers H Pons TL 2000a Leaf respiration of snow gum in the light and dark. Interactions between temperature and irradiance. Plant Physiology 122, 915–923. 10712556 10.1104/pp.122.3.915 PMC58928

[CIT0008] Atkin OK Evans JR Siebke K 1998 Relationship between the inhibition of leaf respiration by light and enhancement of leaf dark respiration following light treatment. Australian Journal of Plant Physiology 25, 437–443.

[CIT0009] Atkin OK Holly C Ball MC 2000b Acclimation of snow gum (*Eucalyptus pauciflora*) leaf respiration to seasonal and diurnal variations in temperature: the importance of changes in the capacity and temperature sensitivity of respiration. Plant, Cell and Environment 23, 15–26.

[CIT0010] Atkin OK Macherel D 2009 The crucial role of plant mitochondria in orchestrating drought tolerance. Annals of Botany 103, 581–597. 18552366 10.1093/aob/mcn094 PMC2707344

[CIT0011] Atkin OK Scheurwater I Pons TL 2007 Respiration as a percentage of daily photosynthesis in whole plants is homeostatic at moderate, but not high, growth temperatures. New Phytologist 174, 367–380. 17388899 10.1111/j.1469-8137.2007.02011.x

[CIT0012] Atkin OK Tjoelker MG 2003 Thermal acclimation and the dynamic response of plant respiration to temperature. Trends in Plant Science 8, 343–351. 12878019 10.1016/S1360-1385(03)00136-5

[CIT0013] Ayub G Smith RA Tissue DT Atkin OK 2011 Impacts of drought on leaf respiration in darkness and light in *Eucalyptus saligna* exposed to industrial-age atmospheric CO_2_ and growth temperature. New Phytologist 190, 1003. 21434926 10.1111/j.1469-8137.2011.03673.x

[CIT0014] Azcón-Bieto J Osmond CB 1983 Relationship between photosynthesis and respiration. The effect of carbohydrate status on the rate of CO_2_ production by respiration in darkened and illuminated wheat leaves. Plant Physiology 71, 574–581. 16662869 10.1104/pp.71.3.574 PMC1066080

[CIT0015] Bartoli CG Gomez F Gergoff G Guiamet J Puntarulo S 2005 Up-regulation of the mitochondrial alternative oxidase pathway enhances photosynthetic electron transport under drought conditions. Journal of Experimental Botany 56, 1269–1276. 15781442 10.1093/jxb/eri111

[CIT0016] Björkman O Badger M Armond PA Turner NC Kramer PJ 1980 Response and adaptation of photosynthesis to high temperatures. Adaptation of plants to water and high temperature stress. New York: John Wiley and Sons, 233–248.

[CIT0017] Bolstad PV Reich P Lee T 2003 Rapid temperature acclimation of leaf respiration rates in *Quercus alba* and *Quercus rubra.* Tree Physiology 23, 969–976. 12952783 10.1093/treephys/23.14.969

[CIT0018] BOM-Australia. 2014 Special Climate Statement 48—one of southeast Australia’s most significant heatwaves. Canberra: Australian Government.

[CIT0019] Bruhn D Mikkelsen TN Atkin OK 2002 Does the direct effect of atmospheric CO_2_ concentration on leaf respiration vary with temperature? Responses in two species of *Plantago* that differ in relative growth rate. Physiologia Plantarum 114, 57–64. 11982935

[CIT0020] Bunce JA 2005 Response of respiration of soybean leaves grown at ambient and elevated carbon dioxide concentrations to day-to-day variation in light and temperature under field conditions. Annals of Botany 95, 1059–1066. 15781437 10.1093/aob/mci117 PMC4246764

[CIT0021] Bunce JA 2007 Direct and acclimatory responses of dark respiration and translocation to temperature. Annals of Botany 100, 67–73. 17483153 10.1093/aob/mcm071 PMC2735291

[CIT0022] Cai T Flanagan LB Syed KH 2010 Warmer and drier conditions stimulate respiration more than photosynthesis in a boreal peatland ecosystem: analysis of automatic chambers and eddy covariance measurements. Plant, Cell and Environment 33, 394–407. 10.1111/j.1365-3040.2009.02089.x 19968825

[CIT0023] Campbell C Atkinson L Zaragoza-Castells J Lundmark M Atkin O Hurry V 2007 Acclimation of photosynthesis and respiration is asynchronous in response to changes in temperature regardless of plant functional group. New Phytologist 176, 375–389. 17692077 10.1111/j.1469-8137.2007.02183.x

[CIT0024] Ciais P Reichstein M Viovy N 2005 Europe-wide reduction in primary productivity caused by the heat and drought in 2003. Nature 437, 529–533. 16177786 10.1038/nature03972

[CIT0025] Cox PM Betts RA Jones CD Spall SA Totterdell IJ 2000 Acceleration of global warming due to carbon-cycle feedbacks in a coupled climate model. Nature 408, 184–187. 11089968 10.1038/35041539

[CIT0026] Crous KY Quentin AG Lin Y-S Medlyn BE Williams DG Barton CVM Ellsworth DS 2013 Photosynthesis of temperate *Eucalyptus globulus* trees outside their native range has limited adjustment to elevated CO_2_ and climate warming. Global Change Biology 19, 3790–3807. 23824839 10.1111/gcb.12314

[CIT0027] Crous KY Zaragoza-Castells J Löw M Ellsworth DS Tissue DT Tjoelker MG Barton CVM Gimeno TE Atkin OK 2011 Seasonal acclimation of leaf respiration in *Eucalyptus saligna* trees: impacts of elevated atmospheric CO_2_ and summer drought. Global Change Biology 17, 1560–1576.

[CIT0028] Davidson A, PrometheusWikicontributors. 2011 Measuring leaf perimeter and leaf area. PrometheusWiki: protocols in ecological & environmental plant physiology, Vol. 2014 Melbourne: PrometheusWiki/CSIRO.

[CIT0029] Downton WJS Berry JA Seemann JR 1984 Tolerance of photosynthesis to high-temperature in desert plants. Plant Physiology 74, 786–790. 16663510 10.1104/pp.74.4.786 PMC1066768

[CIT0030] Duan HL Amthor JS Duursma RA O’Grady AP Choat B Tissue DT 2013 Carbon dynamics of eucalypt seedlings exposed to progressive drought in elevated CO_2_ and elevated temperature. Tree Physiology 33, 779–792. 23963410 10.1093/treephys/tpt061

[CIT0031] Duffy P Tebaldi C 2012 Increasing prevalence of extreme summer temperatures in the U.S. Climatic Change 111, 487–495.

[CIT0032] Faria T Wilkins D Besford RT Vaz M Pereira JS Chaves MM 1996 Growth at elevated CO_2_ leads to down-regulation of photosynthesis and altered response to high temperature in *Quercus suber* L seedlings. Journal of Experimental Botany 47, 1755–1761.

[CIT0033] Flanagan LB Syed KH 2011 Stimulation of both photosynthesis and respiration in response to warmer and drier conditions in a boreal peatland ecosystem. Global Change Biology 17, 2271–2287.

[CIT0034] Flexas J Díaz-Espejo A Berry J Cifre J Galmés J Kaldenhoff R Medrano H Ribas-Carbó M 2007 Analysis of leakage in IRGA’s leaf chambers of open gas exchange systems: quantification and its effects in photosynthesis parameterization. Journal of Experimental Botany 58, 1533–1543. 17339650 10.1093/jxb/erm027

[CIT0035] Flexas J Galmés J Ribas-Carbó M Medrano H Lambers H 2005 The effects of water stress on plant respiration. In: Lambers H Ribas-Carbó M, eds. Plant respiration: from cell to ecosystem. Advances in Photosynthesis and Respiration Volume 18. Dordrecht: Springer, 85–94.

[CIT0036] Forward DF 1960 Effect of temperature on respiration. In: Ruhland W, ed. Encyclopedia of plant physiology, Vol. 12 Berlin: Springer-Verlag, 234–258.

[CIT0037] Funk JL Mak JE Lerdau MT 2004 Stress-induced changes in carbon sources for isoprene production in *Populus deltoides* . Plant, Cell and Environment 27, 747–755.

[CIT0038] Galmés J Ribas-Carbó M Medrano H Flexas J 2007 Response of leaf respiration to water stress in Mediterranean species with different growth forms. Journal of Arid Environments 68, 206–222.

[CIT0039] Gifford RM 1995 Whole plant respiration and photosynthesis of wheat under increased CO_2_ concentration and temperature—long-term vs short- term distinctions for modelling. Global Change Biology 1, 385–396.

[CIT0040] Gifford RM 2003 Plant respiration in productivity models: conceptualisation, representation and issues for global terrestrial carbon-cycle research. Functional Plant Biology 30, 171–186. 10.1071/FP02083 32689003

[CIT0041] Gimeno TE Sommerville KE Valladares F Atkin OK 2010 Homeostasis of respiration under drought and its important consequences for foliar carbon balance in a drier climate: insights from two contrasting *Acacia* species. Functional Plant Biology 37, 323–333.

[CIT0042] Hamerlynck EP Huxman TE Loik ME Smith SD 2000 Effects of extreme high temperature, drought and elevated CO_2_ on photosynthesis of the Mojave desert evergreen shrub, *Larrea tridentata* . Plant Ecology 148, 183–193.

[CIT0043] Hamerlynck EP Knapp AK 1994 Leaf-level responses to light and temperature in two co-occurring *Quercus* (Fagaceae) species: implications for tree distribution patterns. Forest Ecology and Management 68, 149–159.

[CIT0044] Havaux M 1992 Stress tolerance of photosystem II *in vivo*—antagonistic effects of water, heat, and photoinhibition stresses. Plant Physiology 100, 424–432. 16652979 10.1104/pp.100.1.424 PMC1075568

[CIT0045] Havaux M 1993 Rapid photosynthetic adaptation to heat stress triggered in potato leaves by moderately elevated temperatures. Plant, Cell and Environment 16, 461–467.

[CIT0046] Havaux M Greppin H Strasser RJ 1991 Functioning of photosystems I and II in pea leaves exposed to heat stress in the presence or absence of light. Analysis using in-vivo fluorescence, absorbance, oxygen and photoacoustic measurements. Planta 186, 88–98. 24186579 10.1007/BF00201502

[CIT0047] Heskel MA Greaves HE Turnbull MH O’Sullivan OS Shaver GR Griffin KL Atkin OK 2014 Thermal acclimation of shoot respiration in an Arctic woody plant species subjected to 22 years of warming and altered nutrient supply. Global Change Biology 20, 2618–2630. 24510889 10.1111/gcb.12544

[CIT0048] Hsiao TC 1973 Plant responses to water stress. Annual Review of Plant Physiology 24, 519–570.

[CIT0049] Huntingford C Cox PM 2000 An analogue model to derive additional climate change scenarios from existing GCM simulations. Climate Dynamics 16, 586.

[CIT0050] Huntingford C Zelazowski P Galbraith D 2013 Simulated resilience of tropical rainforests to CO_2_-induced climate change. Nature Geoscience 6, 268–273.

[CIT0051] Hüve K Bichele I Ivanova H Keerberg O Pärnik T Rasulov B Tobias M Niinemets Ü 2012 Temperature responses of dark respiration in relation to leaf sugar concentration. Physiologia Plantarum 144, 320–334. 22188403 10.1111/j.1399-3054.2011.01562.x

[CIT0052] Hüve K Bichele I Rasulov B Niinemets Ü 2011 When it is too hot for photosynthesis: heat-induced instability of photosynthesis in relation to respiratory burst, cell permeability changes and H_2_O_2_ formation. Plant, Cell and Environment 34, 113–126. 10.1111/j.1365-3040.2010.02229.x 21029116

[CIT0053] Hüve K Bichele I Tobias M Niinemets Ü 2006 Heat sensitivity of photosynthetic electron transport varies during the day due to changes in sugars and osmotic potential. Plant, Cell and Environment 29, 212–228. 10.1111/j.1365-3040.2005.01414.x 17080637

[CIT0054] IPCC. 2007 Climate change 2007—the physical science basis. Contribution of Working Group I to the Fourth Assessment Report of the Intergovernmental Panel on Climate Change. Cambridge University Press.

[CIT0055] IPCC. 2013 Climate change 2013: the physical science basis. Cambridge University Press.

[CIT0056] James WO 1953 Plant respiration. Oxford: Clarendon Press.

[CIT0057] King AW Gunderson CA Post WM Weston DJ Wullschleger SD 2006 Plant respiration in a warmer world. Science 312, 536–537. 16645083 10.1126/science.1114166

[CIT0058] Knight CA Ackerly DD 2002 An ecological and evolutionary analysis of photosynthetic thermotolerance using the temperature-dependent increase in fluorescence. Oecologia 130, 505–514. 10.1007/s00442-001-0841-0 28547251

[CIT0059] Kruse J Rennenberg H Adams MA 2011 Steps towards a mechanistic understanding of respiratory temperature responses. New Phytologist 189, 659–677. 21223283 10.1111/j.1469-8137.2010.03576.x

[CIT0060] Larcher W 2004 Physiological plant ecology. Ecophysiology and stress physiology of functional groups. Berlin: Springer-Verlag.

[CIT0061] Lawlor DW Cornic G 2002 Photosynthetic carbon assimilation and associated metabolism in relation to water deficits in higher plants. Plant, Cell and Environment 25, 275–294. 10.1046/j.0016-8025.2001.00814.x 11841670

[CIT0062] Lichtenthaler HK 1999 The 1-deoxy- d -xylulose-5-phosphate pathway of isoprenoid biosynthesis in plants. Annual Review of Plant Physiology and Plant Molecular Biology 50, 47–65. 10.1146/annurev.arplant.50.1.47 15012203

[CIT0063] Lippert K Galinski EA 1992 Enzyme stabilization by ectoine-type compatible solutes—protection against heating, freezing and drying. Applied Microbiology and Biotechnology 37, 61–65.

[CIT0064] Loveys BR Atkinson LJ Sherlock DJ Roberts RL Fitter AH Atkin OK 2003 Thermal acclimation of leaf and root respiration: an investigation comparing inherently fast- and slow-growing plant species. Global Change Biology 9, 895–910.

[CIT0065] Loveys BR Scheurwater I Pons TL Fitter AH Atkin OK 2002 Growth temperature influences the underlying components of relative growth rate: an investigation using inherently fast- and slow-growing plant species. Plant, Cell and Environment 25, 975–987.

[CIT0066] Mahecha MD Reichstein M Carvalhais N 2010 Global convergence in the temperature sensitivity of respiration at ecosystem level. Science 329, 838–840. 20603495 10.1126/science.1189587

[CIT0067] Meehl GA Tebaldi C 2004 More intense, more frequent, and longer lasting heat waves in the 21st century. Science 305, 994–997. 15310900 10.1126/science.1098704

[CIT0068] Metcalfe DB Lobo-do-Vale R Chaves MM 2010 Impacts of experimentally imposed drought on leaf respiration and morphology in an Amazon rain forest. Functional Ecology 24, 524–533.

[CIT0069] Mitchell PJ O’Grady AP Tissue DT White DA Ottenschlaeger ML Pinkard EA 2013 Drought response strategies define the relative contributions of hydraulic dysfunction and carbohydrate depletion during tree mortality. New Phytologist 197, 862–872. 23228042 10.1111/nph.12064

[CIT0070] Monson RK Grote R Niinemets Ü Schnitzler J-P 2012 Modeling the isoprene emission rate from leaves. New Phytologist 195, 541–559. 22738087 10.1111/j.1469-8137.2012.04204.x

[CIT0071] Niinemets U 2010 Responses of forest trees to single and multiple environmental stresses from seedlings to mature plants: past stress history, stress interactions, tolerance and acclimation. Forest Ecology and Management 260, 1623–1639.

[CIT0072] Noguchi K Yoshida K 2008 Interaction between photosynthesis and respiration in illuminated leaves. Mitochondrion 8, 87–99. 18024239 10.1016/j.mito.2007.09.003

[CIT0073] O’Sullivan OS Weerasinghe KWLK Evans JR Egerton JJG Tjoelker MG Atkin OK 2013 High-resolution temperature responses of leaf respiration in snow gum (*Eucalyptus pauciflora*) reveal high-temperature limits to respiratory function. Plant, Cell and Environment 36, 1268–1284. 10.1111/pce.12057 23278101

[CIT0074] Ow LF Griffin KL Whitehead D Walcroft AS Turnbull MH 2008 Thermal acclimation of leaf respiration but not photosynthesis in *Populus deltoides×nigra* . New Phytologist 178, 123–134. 18221247 10.1111/j.1469-8137.2007.02357.x

[CIT0075] Penna S 2003 Building stress tolerance through over-producing trehalose in transgenic plants. Trends in Plant Science 8, 355–357. 12927963 10.1016/S1360-1385(03)00159-6

[CIT0076] Peuke AD Gessler A Tcherkez G 2013 Experimental evidence for diel δ^15^N-patterns in different tissues, xylem and phloem saps of castor bean (*Ricinus communis* L.). Plant, Cell and Environment 36, 2219–2228. 10.1111/pce.12132 23663089

[CIT0077] Poorter H Remkes C Lambers H 1990 Carbon and nitrogen economy of 24 wild species differing in relative growth rate. Plant Physiology 94, 621–627. 16667757 10.1104/pp.94.2.621 PMC1077277

[CIT0078] Rahmstorf S Cazenave A Church JA Hansen JE Keeling RF Parker DE Somerville RCJ 2007 Recent climate observations compared to projections. Science 316, 709. 17272686 10.1126/science.1136843

[CIT0079] Rennenberg H Loreto F Polle A Brilli F Fares S Beniwal RS Gessler A 2006 Physiological responses of forest trees to heat and drought. Plant Biology 8, 556–571. 16773557 10.1055/s-2006-924084

[CIT0080] Ribas-Carbó M Taylor NL Giles L Busquets S Finnegan PM Day DA Lambers H Medrano H Berry JA Flexas J 2005 Effects of water stress on respiration in soybean leaves. Plant Physiology 139, 466–473. 16126857 10.1104/pp.105.065565 PMC1203395

[CIT0081] Rodeghiero MIRC Niinemets ULO Cescatti ALES 2007 Major diffusion leaks of clamp-on leaf cuvettes still unaccounted: how erroneous are the estimates of Farquhar *et al*. model parameters? Plant, Cell and Environment 30, 1006–1022. 10.1111/j.1365-3040.2007.001689.x 17617828

[CIT0082] Rodríguez-Calcerrada J Atkin OK Robson TM Zaragoza-Castells J Gil L Aranda I 2010 Thermal acclimation of leaf dark respiration of beech seedlings experiencing summer drought in high and low light environments. Tree Physiology 30, 214–224. 20007131 10.1093/treephys/tpp104

[CIT0083] Rodriguez-Calcerrada J Buatois B Chiche E Shahin O Staudt M 2013 Leaf isoprene emission declines in *Quercus pubescens* seedlings experiencing drought—any implication of soluble sugars and mitochondrial respiration? Environmental and Experimental Botany 85, 36–42.

[CIT0084] Rodríguez-Calcerrada J Jaeger C Limousin JM Ourcival JM Joffre R Rambal S 2011 Leaf CO_2_ efflux is attenuated by acclimation of respiration to heat and drought in a Mediterranean tree. Functional Ecology 25, 983–995.

[CIT0085] Scheurwater I Dunnebacke M Eising R Lambers H 2000 Respiratory costs and rate of protein turnover in the roots of a fast-growing (*Dactylis glomerata* L.) and a slow-growing (*Festuca ovina* L.) grass species. Journal of Experimental Botany 51, 1089–1097. 10948236

[CIT0086] Seemann JR Berry JA Downton WJS 1984 Photosynthetic response and adaptation to high-temperature in desert plants—a comparison of gas-exchange and fluorescence methods for studies of thermal tolerance. Plant Physiology 75, 364–368. 16663627 10.1104/pp.75.2.364 PMC1066913

[CIT0087] Seemann JR Downton WJS Berry JA 1986 Temperature and leaf osmotic potential as factors in the acclimation of photosynthesis to high-temperature in desert plants. Plant Physiology 80, 926–930. 16664743 10.1104/pp.80.4.926 PMC1075231

[CIT0088] Sharkey TD 2005 Effects of moderate heat stress on photosynthesis: importance of thylakoid reactions, rubisco deactivation, reactive oxygen species, and thermotolerance provided by isoprene. Plant, Cell and Environment 28, 269–277.

[CIT0089] Singsaas EL Laporte MM Shi JZ Monson RK Bowling DR Johnson K Lerdau M Jasentuliytana A Sharkey TD 1999 Kinetics of leaf temperature fluctuation affect isoprene emission from red oak (*Quercus rubra*) leaves. Tree Physiology 19, 917–924. 12651303 10.1093/treephys/19.14.917

[CIT0090] Slot M Zaragoza-Castells J Atkin OK 2008 Transient shade and drought have divergent impacts on the temperature sensitivity of dark respiration in leaves of *Geum urbanum* . Functional Plant Biology 35, 1135–1146. 10.1071/FP08113 32688861

[CIT0091] Smith RA Lewis JD Ghannoum O Tissue DT 2012 Leaf structural responses to pre-industrial, current and elevated atmospheric [CO_2_] and temperature affect leaf function in *Eucalyptus sideroxylon* . Functional Plant Biology 39, 285–296. 10.1071/FP11238 32480781

[CIT0092] Sung DY Kaplan F Lee KJ Guy CL 2003 Acquired tolerance to temperature extremes. Trends in Plant Science 8, 179–187. 12711230 10.1016/S1360-1385(03)00047-5

[CIT0093] Taub DR Seemann JR Coleman JS 2000 Growth in elevated CO_2_ protects photosynthesis against high-temperature damage. Plant, Cell and Environment 23, 649–656.

[CIT0094] Tingey DT Lee EH Phillips DL Rygiewicz PT Waschmann RS Johnson MG Olszyk DM 2007 Elevated CO_2_ and temperature alter net ecosystem C exchange in a young Douglas fir mesocosm experiment. Plant, Cell and Environment 30, 1400–1410. 10.1111/j.1365-3040.2007.01713.x 17897410

[CIT0095] Tingley MP Huybers P 2013 Recent temperature extremes at high northern latitudes unprecedented in the past 600 years. Nature 496, 201–205. 23579678 10.1038/nature11969

[CIT0096] Tissue DT Lewis JD 2010 Photosynthetic responses of cottonwood seedlings grown in glacial through future atmospheric CO_2_ vary with phosphorus supply. Tree Physiology 30, 1361–1372. 20884610 10.1093/treephys/tpq077

[CIT0097] Tissue DT Lewis JD Wullschleger SD Amthor JS Griffin KL Anderson R 2002 Leaf respiration at different canopy positions in sweetgum (*Liquidambar styraciflua*) grown in ambient and elevated concentrations of carbon dioxide in the field. Tree Physiology 22, 1157–1166. 12414375 10.1093/treephys/22.15-16.1157

[CIT0098] Tjoelker MG Oleksyn J Lorenc-Plucinska G Reich PB 2009 Acclimation of respiratory temperature responses in northern and southern populations of *Pinus banksiana* . New Phytologist 181, 218–229. 18811616 10.1111/j.1469-8137.2008.02624.x

[CIT0099] Tjoelker MG Oleksyn J Reich PB 1998 Seedlings of five boreal tree species differ in acclimation of net photosynthesis to elevated CO_2_ and temperature. Tree Physiology 18, 715–726. 12651406 10.1093/treephys/18.11.715

[CIT0100] Tjoelker MG Oleksyn J Reich PB 2001 Modelling respiration of vegetation: evidence for a general temperature-dependent *Q* _10_ . Global Change Biology 7, 223–230.

[CIT0101] Tjoelker MG Oleksyn J Reich PB Zytkowiak R 2008 Coupling of respiration, nitrogen, and sugars underlies convergent temperature acclimation in *Pinus banksiana* across wide-ranging sites and populations. Global Change Biology 14, 782–797.

[CIT0102] Valladares F Gianoli E Gomez JM 2007 Ecological limits to plant phenotypic plasticity. New Phytologist 176, 749–763. 17997761 10.1111/j.1469-8137.2007.02275.x

[CIT0103] Velikova V Várkonyi Z Szabó M 2011 Increased thermostability of thylakoid membranes in isoprene-emitting leaves probed with three biophysical techniques. Plant Physiology 157, 905–916. 21807886 10.1104/pp.111.182519 PMC3192565

[CIT0104] Vu JCV Newman YC Allen LH Gallo-Meagher M Zhang MQ 2002 Photosynthetic acclimation of young sweet orange trees to elevated growth CO_2_ and temperature. Journal of Plant Physiology 159, 147–157.

[CIT0105] Wani SH Singh NB Haribhushan A Mir JI 2013 Compatible solute engineering in plants for abiotic stress tolerance—role of glycine betaine. Current Genomics 14, 157–165. 24179438 10.2174/1389202911314030001 PMC3664465

[CIT0106] Weerasinghe LK Creek D Crous KY Xiang S Liddell MJ Turnbull MH Atkin OK 2014 Canopy position affects the relationships between leaf respiration and associated traits in a tropical rainforest in Far North Queensland. Tree Physiology 34, 564–584. 24722001 10.1093/treephys/tpu016

[CIT0107] Whitehead D Griffin KL Turnbull MH Tissue DT Engel VC Brown KJ Schuster WSF Walcroft AS 2004 Response of total night-time respiration to differences in total daily photosynthesis for leaves in a *Quercus rubra* L. canopy: implications for modelling canopy CO_2_ exchange. Global Change Biology 10, 925–938.

[CIT0108] Wise RR Olson AJ Schrader SM Sharkey TD 2004 Electron transport is the functional limitation of photosynthesis in field-grown Pima cotton plants at high temperature. Plant, Cell and Environment 27, 717–724.

[CIT0109] Wullschleger SD Norby RJ Gunderson CA 1992 Growth and maintenance respiration in leaves of *Liriodendron tulipifera* L. exposed to long-term carbon dioxide enrichment in the field. New Phytologist 121, 515–523.

[CIT0110] Wythers KR Reich PB Bradford JB 2013 Incorporating temperature-sensitive Q_10_ and foliar respiration acclimation algorithms modifies modeled ecosystem responses to global change. Journal of Geophysical Research: Biogeosciences 118, 77–90.

[CIT0111] Wythers KR Reich PB Tjoelker MG Bolstad PB 2005 Foliar respiration acclimation to temperature and temperature variable *Q* _10_ alter ecosystem carbon balance. Global Change Biology 11, 435–449.

[CIT0112] Xu CY Salih A Ghannoum O Tissue DT 2012 Leaf structural characteristics are less important than leaf chemical properties in determining the response of leaf mass per area and photosynthesis of *Eucalyptus saligna* to industrial-age changes in CO_2_ and temperature. Journal of Experimental Botany 63, 5829–5841. 22915750 10.1093/jxb/ers231

[CIT0113] Zagdanska B 1995 Respiratory energy demand for protein turnover and ion transport in wheat leaves upon water deficit. Physiologia Plantarum 95, 428–436.

[CIT0114] Zaragoza-Castells J Sanchez-Gomez D Hartley IP Matesanz S Valladares F Lloyd J Atkin OK 2008 Climate-dependent variations in leaf respiration in a dry-land, low productivity Mediterranean forest: the importance of acclimation in both high-light and shaded habitats. Functional Ecology 22, 172–184.

[CIT0115] Zha T Wang KY Ryyppo A Kellomaki S 2002 Needle dark respiration in relation to within-crown position in Scots pine trees grown in long-term elevation of CO_2_ concentration and temperature. New Phytologist 156, 33–41.

