# Supplementary material for: Drought increases heat tolerance of leaf respiration in Eucalyptus globulus saplings grown under both ambient and elevated atmospheric [CO2] and temperature
Source: J Exp Bot. 2014 Sep 9;65(22):6471–85. doi: 10.1093/jxb/eru367 (PMC4246183; doi:10.1093/jxb/eru367)
Supplement: Supplementary Data [file supp_65_22_6471__index.html]

Drought increases heat tolerance of leaf respiration in Eucalyptus globulus saplings grown under both ambient and elevated atmospheric [CO2] and temperature — Drought increases heat tolerance of leaf respiration in Eucalyptus globulus saplings grown under both ambient and elevated atmospheric [CO2] and temperature — Supplementary Data 

# Drought increases heat tolerance of leaf respiration in *Eucalyptus globulus* saplings grown under both ambient and elevated atmospheric [CO2] and temperature

## Supplementary Data

Data files

**Files in this Data Supplement:**

- Supplementary Data - Supplementary Data
