# Supplementary material for: Drought increases heat tolerance of leaf respiration in Eucalyptus globulus saplings grown under both ambient and elevated atmospheric [CO2] and temperature
Source: J Exp Bot. 2014 Sep 9;65(22):6471–85. doi: 10.1093/jxb/eru367 (PMC4246183; doi:10.1093/jxb/eru367)

Drought increases heat tolerance of leaf respiration in *Eucalyptus globulus* saplings grown under both ambient and elevated atmospheric [CO_2] and temperature

Paul P G Gauthier, Kristine Y Crous, Gohar Ayub, Honglang Duan, Lasantha K Weerasinghe, David S Ellsworth, Mark G Tjoelker, John R Evans, David T Tissue, and Owen K Atkin

Supplementary Data

Supplementary Table S1. 3-way ANOVA of leaf light-saturated net photosynthesis per unit area (A_{sat}), stomatal conductance (g_s), internal CO_2 concentration (C_i) and the ratio between dark respiration and A_{sat} (R_{dark}:A_{sat}) for the second week of the first drought phase (week 4) and the first week of the second drought phase (week 6). Main factors used in the analysis were atmospheric growth CO_2 concentration (CO_2), growth temperature (*T*) and water availability (H_2O). *P* values in **bold** indicate significant effects ($P < 0.05$). See Figure S4 for trait values of A_{sat}, Figure S2 for traits values of g_s, Figure S3 for C_i and Figure S6 for R_{dark}:A_{sat}.

Source (growth environment)	d.f	Week 4				Week 6			
		A_{sat}	g_s	C_i	$R_{dark}:A_{sat}$	A_{sat}	g_s	C_i	$R_{dark}:A_{sat}$
H_2O	1	**<0.001**	**<0.001**	**<0.001**	**0.027**	**<0.001**	**<0.001**	**<0.001**	**0.001**
T	1	0.093	0.268	0.825	0.816	0.109	0.126	**<0.001**	.065
CO_2	1	0.088	0.097	**<0.001**	0.122	0.632	0.112	0.980	.994
H_2O * *T*	1	0.867	0.321	0.623	0.769	0.183	0.227	**<0.001**	.408
H_2O * CO_2	1	0.536	0.122	**0.005**	0.171	0.149	0.096	.968	.415
T * CO_2	1	0.986	0.815	0.870	0.793	0.829	0.866	.400	.353
H_2O * *T* * CO_2	1	0.662	0.840	0.867	0.763	0.438	0.958	.480	.527
Error	16								

Figure S1. Week-to-week variations (commencing in mid Nov 2010) in the prevailing late-morning/early-afternoon leaf temperature (*T*) during leaf gas exchange exchange measurements of *Eucalyptus globulus* (n = 3 ± s.e.). Data shown are the averages (± s.e.) for plants grown under two growth *T* scenarios (ambient and ambient+3 ^{o}C), with values being the average of all measurements made within each growth *T* environment, irrespective of atmospheric [CO_2] or water availability.

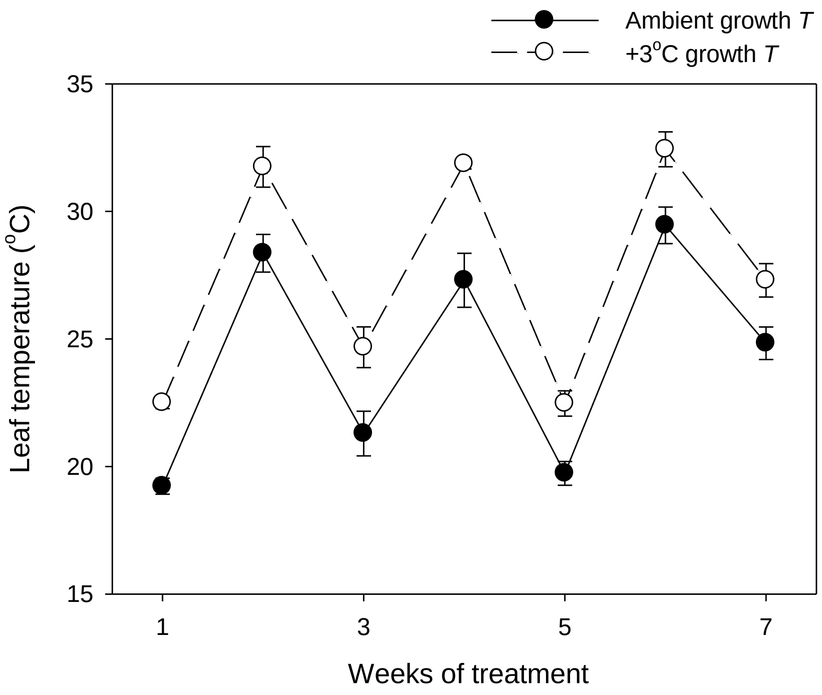

Figure S2. Effect of periodic drought on mean values (*n* = 3 ± s.e.) of stomatal conductance (g_s) of *Eucalyptus globulus* fully-expanded leaves (measured over several weeks commencing early-November 2010) for plants grown under two atmospheric [CO_2] (400 and 640 μmol mol^{-1} for ambient and elevated [CO_2], respectively) and two growth temperature (*T*, ambient and ambient+3 ^{o}C) scenarios: (A) ambient [CO_2] and ambient *T*; (B) elevated [CO_2] and ambient *T*; (C) ambient [CO_2] and elevated *T*; and (D) elevated [CO_2] and elevated *T*. Within each [CO_2]-growth *T* combination, values are shown for well watered (closed symbols) and drought-treated (open symbols). The shaded regions designate two periods of controlled drought, with the intervening non-shaded region indicating when drought treated plants were re-watered after the first drought period. Significant student t-test *P* values of comparisons between drought and well-watered g_s values are indicated with * for P<0.1, ** for P<0.05 and *** for P<0.01.

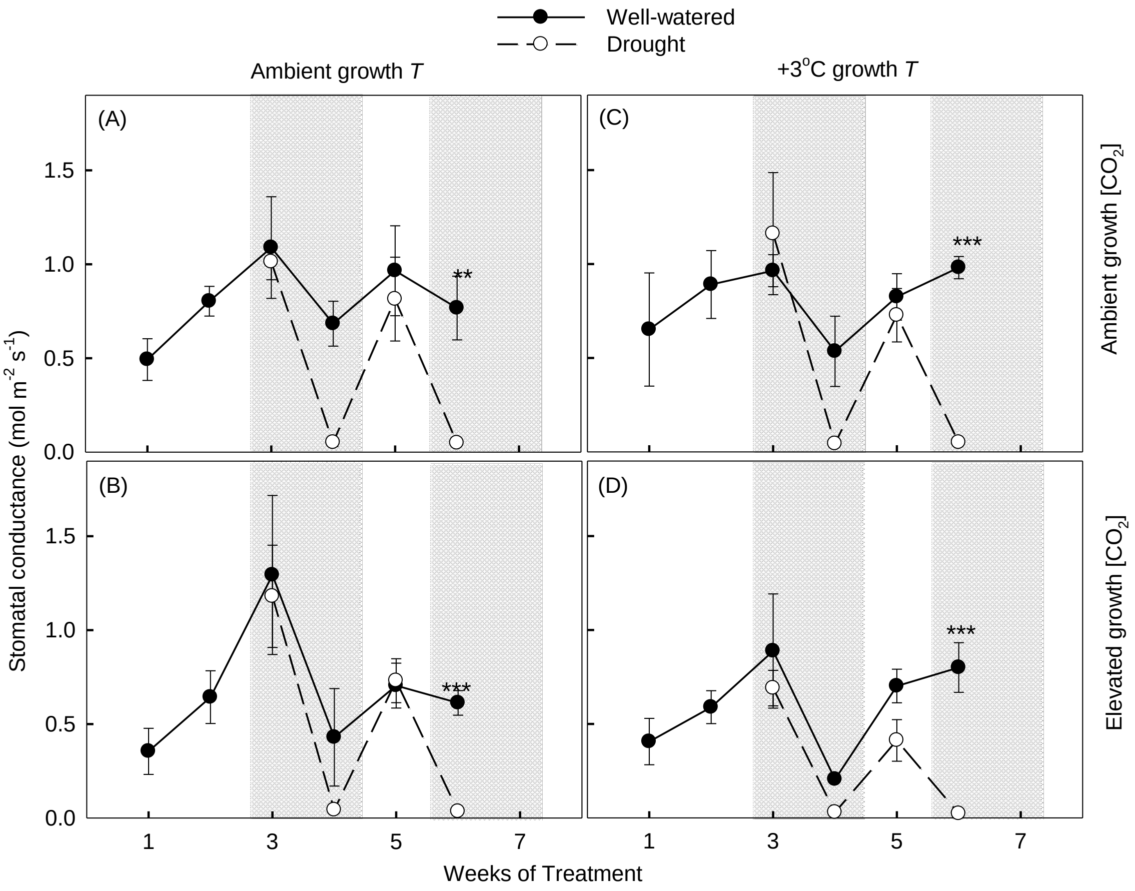

Figure S3. Effect of periodic drought on mean values (n = 3 ± s.e.) of internal CO_2 concentration (c_i) measured under light-saturating conditions for *Eucalyptus globulus* fully-expanded leaves (measured over several weeks commencing early-November 2010) for plants grown under two atmospheric [CO_2] (400 and 640 μmol mol^{-1} for ambient and elevated [CO_2], respectively) and two growth temperature (T, ambient and ambient+3 ^{o}C) scenarios: (A) ambient [CO_2] and ambient T; (B) elevated [CO_2] and ambient T; (C) ambient [CO_2] and elevated T; and (D) elevated [CO_2] and elevated T. Within each [CO_2]-growth T combination, values are shown for well watered (closed symbols) and drought-treated (open symbols). The shaded regions designate two periods of controlled drought, with the intervening non-shaded region indicating when drought treated plants were re-watered after the first drought period.

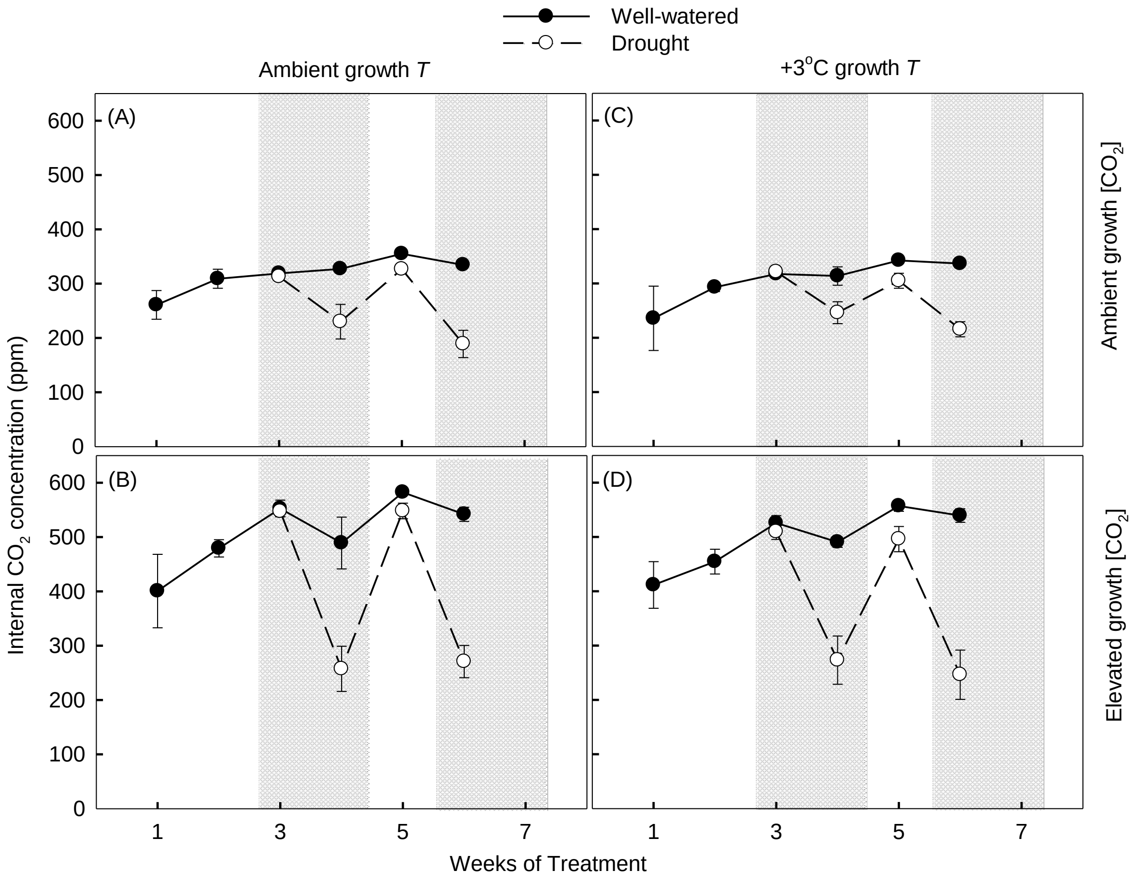

Figure S4. Effect of periodic drought on mean values (*n* = 3 ± s.e.) of light-saturated photosynthesis (A_{sat}) of *Eucalyptus globulus* fully-expanded leaves (measured over several weeks commencing early-November 2010) for plants grown under two atmospheric [CO_2] (400 and 640 μmol mol^{-1} for ambient and elevated [CO_2], respectively) and two growth temperature (*T*, ambient and ambient +3 ^{o}C) scenarios: (A) ambient [CO_2] and ambient *T*; (B) elevated [CO_2] and ambient *T*; (C) ambient [CO_2] and elevated *T*; and (D) elevated [CO_2] and elevated *T*. Within each [CO_2]-growth *T* combination, values are shown for well watered (closed symbols) and drought-treated (open symbols). The shaded regions designate two periods of controlled drought, with the intervening non-shaded region indicating when drought treated plants were re-watered after the first drought period. Significant student t-test *P* values of comparisons between drought and well-watered A_{sat} values are indicated with * for *P*<0.1, ** for *P*<0.05 and *** for *P*<0.01.

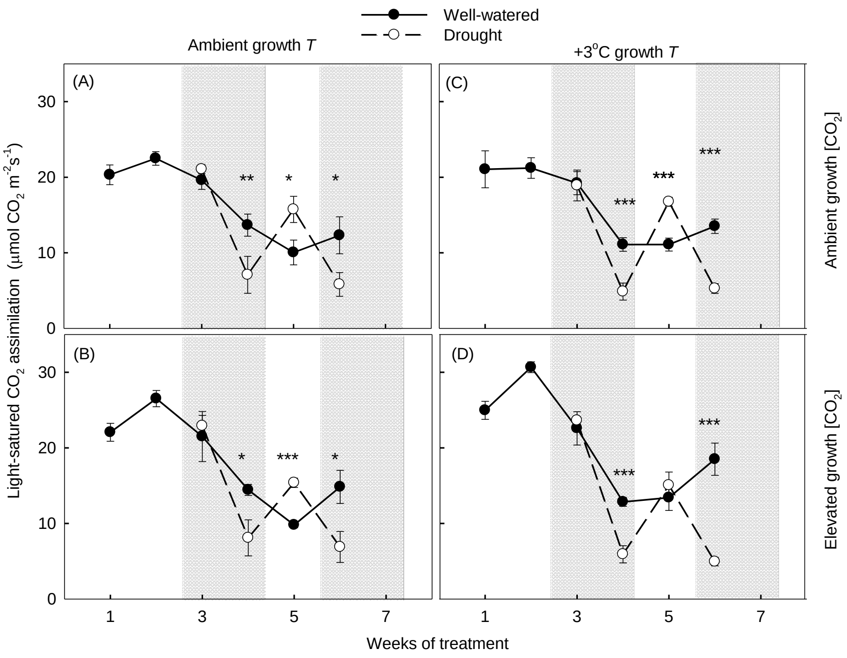

Figure S5. Effect of periodic drought on mean values (*n* = 3 ± s.e.) of leaf respiration in darkness (R_{dark}) of *Eucalyptus globulus* fully-expanded leaves (measured over several weeks commencing early-November 2010) for plants grown under two atmospheric [CO_2] (400 and 640 μmol mol^{-1} for ambient and elevated [CO_2], respectively) and two growth temperature (*T*, ambient and ambient+3 ^{o}C) scenarios: (A) ambient [CO_2] and ambient *T*; (B) elevated [CO_2] and ambient *T*; (C) ambient [CO_2] and elevated *T*; and (D) elevated [CO_2] and elevated *T*. Within each [CO_2]-growth *T* combination, values are shown for well watered (closed symbols) and drought-treated (open symbols). The shaded regions designate two periods of controlled drought, with the intervening non-shaded region indicating when drought plants were re-watered after the first drought period.

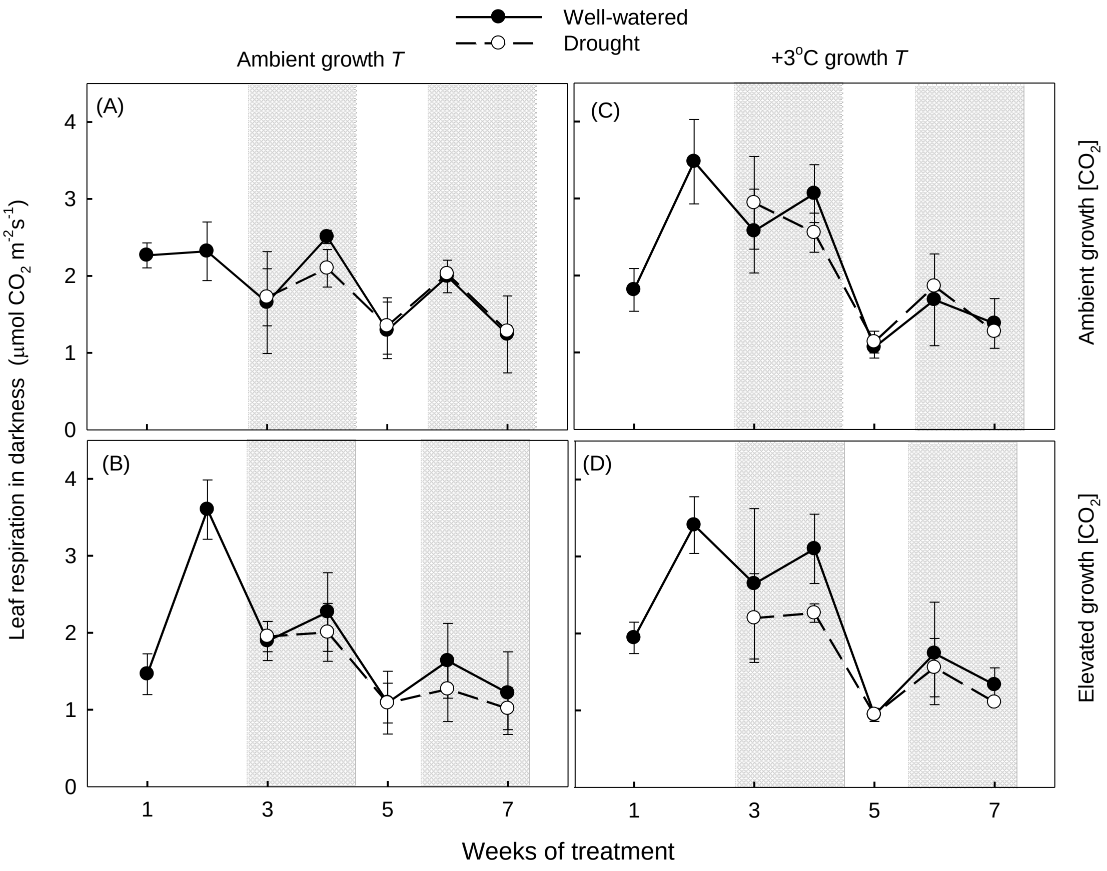

Figure S6. Effect of periodic drought on mean values (*n* = 3 ± s.e.) of the ratio of leaf respiration in darkness to light-saturated photosynthesis (i.e. R_{dark}/A_{sat}) of *Eucalyptus globulus* fully-expanded leaves (measured over several weeks commencing early-November 2010) for plants grown under two atmospheric [CO_2] (400 and 640 μmol mol^{-1} for ambient and elevated [CO_2], respectively) and two growth temperature (*T*, ambient and ambient+3 ^{o}C) scenarios: (A) ambient [CO_2] and ambient *T*; (B) elevated [CO_2] and ambient *T*; (C) ambient [CO_2] and elevated *T*; and (D) elevated [CO_2] and elevated *T*. Within each [CO_2]-growth T combination, values are shown for well watered (closed symbols) and drought-treated (open symbols). The shaded regions designate two periods of controlled drought, with the intervening non-shaded region indicating when drought plants were re-watered after the first drought period. Significant student t-test *P* values of comparisons between drought and well-watered R_{dark}/A_{sat} values are indicated with * for $P<0.1$, ** for $P<0.05$ and *** for $P<0.01$.

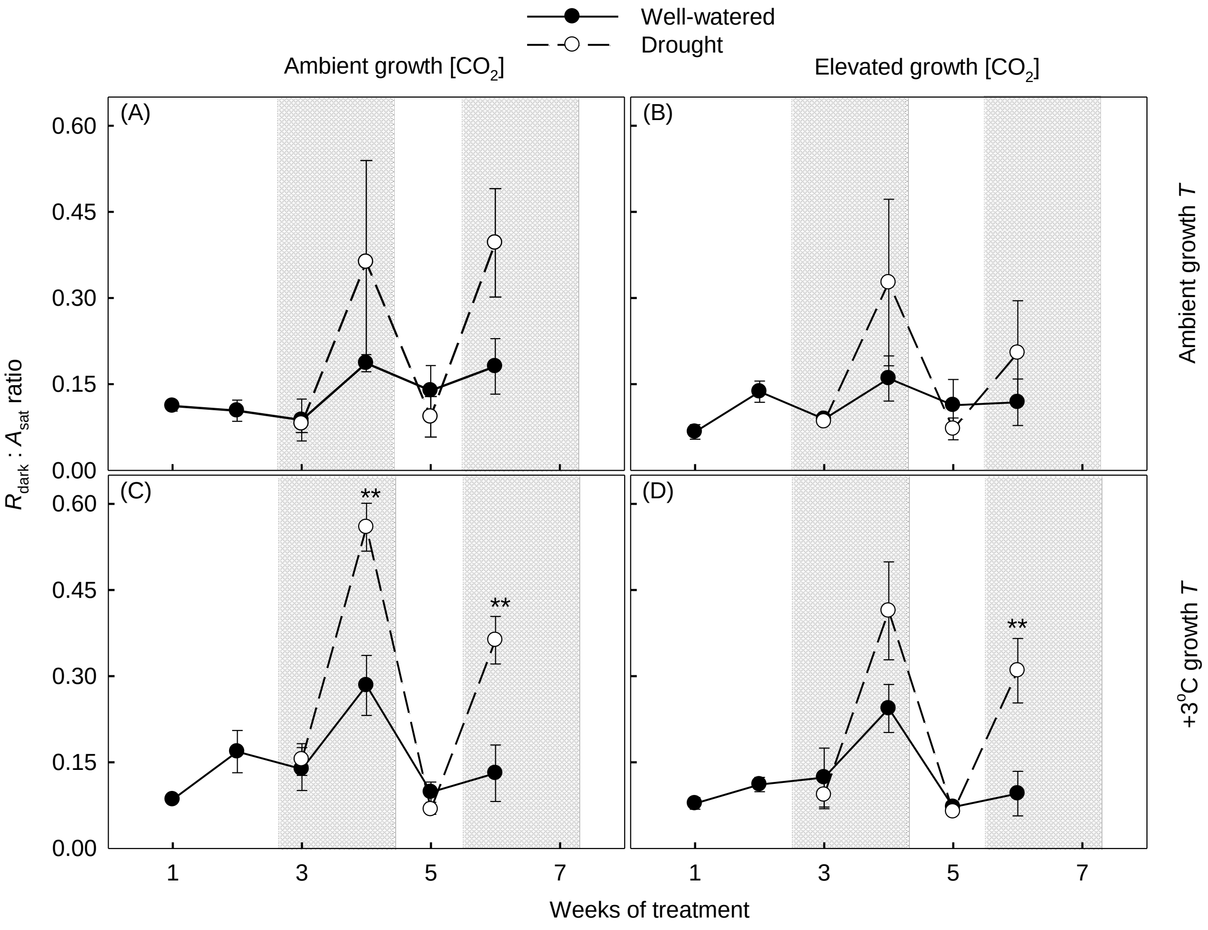

Supplement: Supplementary Data [file supp_eru367_jexbot126334_file001.pdf]
